# Prenatal Exposure to Lipopolysaccharide or Valproate Leads to Abnormal Accumulation of the NMDA Receptor Agonist D‐Aspartate in the Adolescent Rat Brain

**DOI:** 10.1111/jnc.70095

**Published:** 2025-05-28

**Authors:** Anna Di Maio, Isar Yahyavi, Valeria Buzzelli, Zoraide Motta, Fabrizio Ascone, Lorenza Putignani, Alessandro Usiello, Loredano Pollegioni, Viviana Trezza, Francesco Errico

**Affiliations:** ^1^ CEINGE Biotecnologie Avanzate “Franco Salvatore” Naples Italy; ^2^ Department of Environmental, Biological and Pharmaceutical Sciences and Technologies Università degli Studi della Campania “Luigi Vanvitelli” Caserta Italy; ^3^ Department of Science Roma Tre University Rome Italy; ^4^ “The Protein Factory 2.0”, Dipartimento di Biotecnologie e Scienze della Vita Università degli Studi dell'insubria Varese Italy; ^5^ Unit of Microbiomics and Unit of Research of Microbiome Bambino Gesù Children's Hospital, IRCCS Rome Italy; ^6^ Department of Life Science, Health, and Health Professions, Link Campus University Rome Italy; ^7^ Neuroendocrinology, Metabolism and Neuropharmacology Unit IRCCS Fondazione Santa Lucia Rome Italy; ^8^ Dipartimento di Agraria Università degli Studi di Napoli “Federico II” Portici Italy

**Keywords:** autism spectrum disorder, D‐aspartate, D‐serine, lipopolysaccharide, NMDA receptor, valproate

## Abstract

Autism spectrum disorder (ASD) is a neurodevelopmental psychiatric condition linked to glutamatergic neurotransmission disruption. Although endogenous D‐serine and D‐aspartate modulate glutamatergic N‐methyl D‐aspartate receptor (NMDAR) activity, their involvement in ASD remains elusive. We measured the levels of D‐aspartate, D‐serine, and other key neuroactive amino acids, and their direct precursors in brain regions, plasma, and feces of environmental ASD rat models prenatally exposed to lipopolysaccharide or valproate, both during adolescence and early adulthood, as well as in a genetic ASD model, the *Fmr1‐^Δ^exon8* rat. No significant changes were found in plasma and feces. Conversely, we observed a prominent accumulation of D‐aspartate in several brain regions of lipopolysaccharide‐ and valproate‐exposed rats, selectively during adolescence, while D‐serine level variations were more limited. No significant amino acid changes were observed in the *Fmr1‐^Δ^exon8* rat brain. We also assayed the activity of the main enzymes involved in cerebral D‐serine and D‐aspartate metabolism, suggesting that their regulation extends beyond their metabolic enzymes. These findings highlight that prenatal environmental stressors disrupt D‐amino acid levels selectively in ASD rat brains, emphasizing the role of early NMDAR dysfunction in ASD‐related phenotypes.

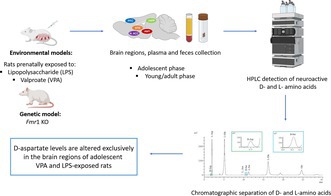

## Graphical abstract

Autism spectrum disorder (ASD) is a neurodevelopmental condition linked to glutamatergic neurotransmission abnormalities. We analyzed by HPLC the cerebral and peripheral profiles of D‐aspartate, D‐serine, and other L‐amino acids involved in NMDA receptor signaling in environmental ASD rat models prenatally exposed to lipopolysaccharide or valproate, during adolescence and early adulthood, and in the genetic Fmr1‐^Δ^exon8 rat model. Prenatal lipopolysaccharide or valproate exposure led to a region‐specific D‐aspartate accumulation in adolescent brains, with no changes in plasma or feces, indicating brain‐selective metabolic disruption. D‐serine changes were modest; no alterations were found in the genetic model. These results highlight the impact of early environmental stressors on D‐aspartate metabolism, supporting its involvement in ASD‐related neurodevelopmental changes.

**Abbreviations**
ASD: autism spectrum disorder
DAAO: D‐amino acid oxidase
D‐Asp: D‐aspartate
DASPO: D‐aspartate oxidase
DDO: D‐aspartate oxidase
D‐Ser: D‐serine
FAD: flavin adenine dinucleotide
*Fmr1*
: *Fragile X messenger ribonucleoprotein 1*
Gly: glycine
HPLC: high‐performance liquid chromatography
L‐Asn: L‐asparagine
L‐Asp: L‐aspartate
LDH: lactate dehydrogenase
L‐Gln: L‐glutamine
L‐Glu: L‐glutamate
LPS: lipopolysaccharide
L‐Ser: L‐serine
MIA: maternal immune activation
NMDAR: N‐methyl D‐Aspartate receptor
PBS: phosphate‐buffered saline
PFC: prefrontal cortex
PolyI:C: polyriboinosinic–polyribo‐cytidilic acid
SR: serine racemase
TCA: trichloroacetic acid
VPA: valproate
WT: wild‐type

## 1 Introduction

Autism spectrum disorder (ASD) encompasses a range of neurodevelopmental psychiatric conditions typically diagnosed in early childhood, characterized by deficits in social interaction and communication, difficulties in sustaining relationships, and the presence of restricted, repetitive behaviors and interests (Tchaconas and Adesman 2013). The precise cause of ASD remains unclear, arising from a complex interplay between genetic and environmental factors. Recent research has focused on the contribution of maternal environmental stressors to ASD risk (Ornoy et al. 2015; Love et al. 2024). Among such factors, maternal immune activation (MIA) caused by exposure to pathogens or inflammation (Hall et al. 2023; Gardner et al. 2024) and prenatal exposure to the mood stabilizer and antiepileptic drug valproic acid (VPA) (Christensen et al. 2013; Bromley et al. 2013) during critical periods of gestation have been related to an increased susceptibility to develop various psychiatric and neurological disorders in the offspring, including ASD. Based on this clinical evidence, prenatal exposure to infectious agents such as the endotoxin lipopolysaccharide (LPS) or maternal treatment with VPA are widely used in the preclinical setting to induce in rodents behavioral and neural anomalies resembling those observed in ASD (Ornoy et al. 2024; Tartaglione et al. 2019; Zarate‐Lopez et al. 2024; Carbone et al. 2023).

Although the exact etiology of ASD remains undefined, disruptions in the glutamatergic neurotransmission system have been proposed as a potential pathophysiological mechanism (Burnashev and Szepetowski 2015), given its essential role in brain development, synaptic plasticity, and behavior, including cognitive functions (Jansson and Akerman 2014; Volk et al. 2015). Accordingly, various studies highlight that both LPS and VPA exposure are linked to glutamatergic neurotransmission abnormalities in the offspring brain (Bergdolt and Dunaevsky 2019; Nicolini and Fahnestock 2018).

In addition to the canonical excitatory amino acids, two endogenous amino acids in the atypical D‐configuration—D‐serine (D‐Ser) and D‐aspartate (D‐Asp)—are well recognized for their pharmacological ability to stimulate glutamatergic transmission at the NMDA receptor (NMDAR) site. Specifically, D‐Ser is the primary co‐agonist of NMDARs at central excitatory synapses (Wolosker and Radzishevsky 2013; Mothet et al. 2015), while D‐Asp acts as an agonist of both NMDARs and metabotropic mGlu5 receptors (Errico et al. 2020). Both D‐amino acids are present at high levels in the developing brain, with D‐Ser remaining consistently high throughout life, while D‐Asp drastically declines after birth (Hashimoto et al. 1993; Punzo et al. 2016; De Rosa et al. 2020). The decline in D‐Asp coincides with the onset of D‐aspartate oxidase (DDO or DASPO) activity, the enzyme responsible for its degradation (Van Veldhoven et al. 1991; Katane and Homma 2010; Molla et al. 2020).

Although both D‐amino acids play key roles in NMDAR activity and function, D‐Asp involvement has been less extensively studied compared to D‐Ser (Souza et al. 2023; Pollegioni and Sacchi 2010; Errico et al. 2020). Specifically, elevated levels of D‐Asp enhance NMDAR‐mediated synaptic plasticity, dendritic growth, and cortical activity in adult mice and rats (Errico et al. 2014, 2008; Kitamura et al. 2019). Conversely, D‐Asp depletion in the developing brain of knockin mice with additional *Ddo* gene copy results in altered corticogenesis, reduced corticostriatal gray matter volume, increased number of cortical GABAergic interneurons, and deficits in cognitive and social recognition abilities in adulthood (Lombardo et al. 2022; Grimaldi et al. 2021; De Rosa et al. 2020). Consistent with a neurodevelopmental influence of D‐Asp metabolism in regulating brain development, a clinical case of ASD and intellectual disability was reported in a patient with a chromosomal duplication encompassing the entire *DDO* gene (Lombardo et al. 2022).

**FIGURE 1:**
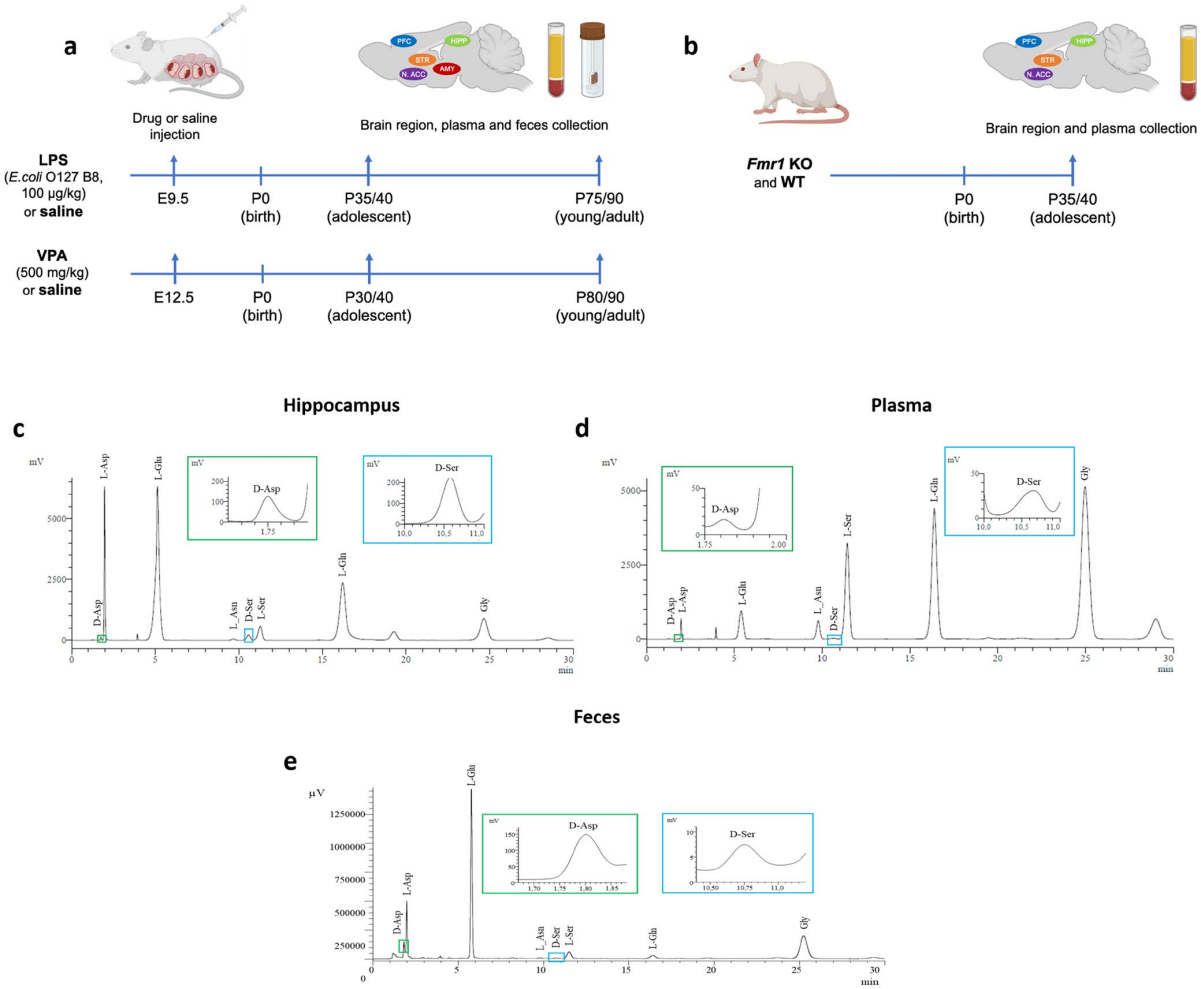
Schematic overview of rat model generation and experimental workflow. (a, b) Experimental timeline for (a) environmental and (b) genetic rat model development, including sample collection from brain regions, plasma and feces at adolescence and young adulthood. These samples were analyzed using HPLC to assess amino acid levels and enzymatic activity assays. Image created with BioRender.com (www.biorender.com). (c–e) Representative HPLC chromatograms from (c) the hippocampus (as an example of a brain region), (d) plasma and (e) fecal samples. Each chromatogram highlights the specificity of each amino acid peak, identified based on retention times and peak areas, compared with internal standards (see Section 2).

Despite the growing interest in D‐Asp and D‐Ser metabolism alterations in schizophrenia pathophysiology (Wolosker and Radzishevsky 2013; Labrie et al. 2012; Coyle et al. 2020; De Rosa et al. 2022; Nuzzo et al. 2017; Errico et al. 2013; Garofalo et al. 2024), the role of these D‐amino acids has not been comprehensively explored in the context of ASD, either in patients or animal models. In the present work, we performed chiral high‐performance liquid chromatography (HPLC) analysis on plasma, feces, and various brain regions of LPS‐ and VPA‐exposed rats during adolescence and early adulthood to profile the levels of D‐Asp, D‐Ser, and the major amino acids involved in glutamatergic neurotransmission. In addition to environmental ASD models, we also performed the same neurochemical analysis in adolescent rats with a genetic deletion of *Fragile X messenger ribonucleoprotein 1* (*Fmr1*) (*Fmr1‐^Δ^exon 8* rats) (Figure 1b), which causes Fragile X Syndrome, the most common inherited form of ASD (Richter and Zhao 2021). Finally, we systematically determined the activity of the enzymes involved in D‐amino acid metabolism, that is, serine racemase (SR) for the synthesis and D‐amino acid oxidase (DAAO) and DASPO for the degradation side. Our findings highlight that the prenatal environmental stressors, VPA and LPS, elicit dramatic perturbations in cerebral D‐amino acid levels in rat offspring during adolescence, suggesting that dysmetabolism of these endogenous NMDAR ligands plays a role in modulating ASD‐related phenotypes.

## 2 Methods

### 2.1 Animals and Experimental Design of the Study

To generate the LPS and VPA environmental models of ASD, female Wistar rats (Strain Code 003; Charles River Laboratories, Calco, Italy), weighing 250 ± 15 g, were mated overnight. The morning when spermatozoa were found was designated as gestational day 1. Pregnant rats received a single intraperitoneal (i.p.) injection of LPS (100 μg/kg) (from *Escherichia coli* , serotype O127:B8, Sigma Aldrich‐Merck, Darmstadt, Germany. Cat. no.: 35739) or VPA (500 mg/kg) (Cayman Chemical, Ann Arbor, MI, USA. Cat. no.: L3129) at embryonic day 9.5 (E9.5) or E12.5, respectively (Carbone et al. 2023; Servadio et al. 2016). Control females received a single i.p. injection of saline.

Pregnant rats from all experimental groups were singly housed in Macrolon cages (40 (length) × 26 (width) × 20 (height) cm), under controlled conditions (temperature 20°C–21°C, 55%–65% relative humidity and 12/12 h light cycle with lights on at 07:00 h). Food and water were available ad libitum.

Newborn litters found up to 17.00 h were considered to be born on that day (postnatal day 0, P0). On P1, the litters were culled to eight animals (six males and two females) to reduce the litter size‐induced variability in the growth and development of pups during the postnatal period (Agnish and Keller 1997). On P21, the pups were weaned and housed in groups of three with cage mates belonging to the same experimental group. No randomization was performed to allocate animals to experimental groups. No exclusion criteria were pre‐determined before the beginning of the study. One pup per litter, from different litters per treatment group (LPS‐, VPA‐ and relative saline‐treated rats), was randomly selected and sacrificed via decapitation at adolescence (P35–40) or early adulthood (P75–90). Plasma and brain regions, including the PFC, hippocampus, dorsal striatum, nucleus accumbens, and amygdala, were collected for biochemical analyses (*n* = 6 rats/treatment/age) (Figure 1a). Given that ASD is a neurodevelopmental disorder, these specific time points were chosen to capture critical phases of brain maturation, thereby offering a more comprehensive view of its underlying pathophysiology. To eliminate potential confounding factors, animals selected for biochemical assays were behaviorally naïve (i.e., they did not perform any behavioral test). This strategy ensured that neurochemical outcomes remained unaffected by prior behavioral manipulations, preserving the integrity of the biochemical data.

Plasma and brain regions were also collected from adolescent (P35‐40) *Fmr1‐^Δ^exon 8* rats (Horizon Discovery, formerly SAGE Labs, USA) and their wild‐type (WT) controls (*n* = 6 rats/genotype) (Figure 1b), which have been validated as a genetic model of ASD (Rava et al. 2025; Schiavi et al. 2023, 2022). Another set of LPS‐ or VPA‐treated rats and relative controls (*n* = 10 rats/treatment/age) were used for feces withdrawal at adolescence (P30–40) or early adulthood (P80–90) (Figure 1a).

Across all experimental conditions and time points, a total of 140 animals were used in this study. The minimum number of animals and sample sizes required to achieve statistical significance were determined by power analysis and prior experience, assuming 80% power at a significance level of 0.05 (G*Power 3.1 software).

The experiments were performed in agreement with the ARRIVE (Animals in Re‐search: Reporting In Vivo Experiments) guidelines (Kilkenny et al. 2010), the guidelines of the Italian Ministry of Health (D.L. 26/14) and the European Community Directive 2010/63/EU and were approved by the Italian Ministry of Health (authorization numbers: 31/2019‐PR, 1207/2016‐PR and 65/2023‐PR).

### 2.2 HPLC Analysis of Amino Acid Content

Feces were homogenized in 1:20 (w/v) phosphate‐buffered saline (PBS), sonicated (3 cycles, 20 s/cycle), incubated on ice for 15 min, and centrifuged at 4°C at 12 100 × g for 5 min (Gonda et al. 2023). Supernatants were mixed in a 1:10 dilution with HPLC‐grade methanol and centrifuged at 13 000 × g for 10 min. Similarly, plasma was mixed in a 1:10 dilution with HPLC‐grade methanol and centrifuged at 13 000 × g for 10 min (Imarisio et al. 2024). Plasma and feces supernatants were dried and then suspended in 0.2 M trichloroacetic acid (TCA). Brain samples were homogenized in 1:20 (w/v) 0.2 M TCA, sonicated (3 cycles, 10 s/cycle) and centrifuged at 13 000 × g for 20 min. The protein pellets were stored at −80°C for protein quantification (Serra et al. 2023). TCA supernatants deriving from plasma, feces, or brain samples were neutralized with NaOH and subjected to precolumn derivatization with o‐phthaldialdehyde/N‐acetyl‐l‐cysteine. Amino acid derivatives were resolved on a UHPLC Nexera X3 system (Shimadzu) by using a Shim‐pack GIST C18 3‐μm reversed‐phase column (Shimadzu, 4.0 × 150 mm) under isocratic conditions (0.1 M sodium acetate buffer, pH 6.2, 1% tetrahydrofuran, and 1 mL/min ow rate). A washing step in 0.1 M sodium acetate buffer, 3% tetrahydrofuran, and 47% acetonitrile was performed after every run. Identification and quantification of amino acids were based on retention times and peak areas, compared with those associated with internal standards (Figure 1c–e). Total protein content of brain sample homogenates was determined using the Bradford assay method after resolubilizing the TCA‐precipitated protein pellets. The total content of amino acids detected in plasma samples was expressed as μM; in feces homogenates, it was normalized to the fecal weight and expressed as nmol/g of feces, while in brain homogenates, it was normalized to the total protein content and expressed as nmol/mg protein. D‐Asp/total Asp and D‐Ser/total Ser ratios were expressed as percentages (%) while the L‐Gln/L‐Gln ratio was expressed as an absolute value.

### 2.3 Activity Assay

Tissue samples were resuspended (10 mg of tissue in 200 μL of buffer) in 20 mM sodium phosphate pH 8.0, 0.1% Triton X‐100 (93 426, Fluka) added of complete protease‐ (11836153001, Roche) and phosphatase‐inhibitors (5870, Cell Signaling) cocktails following the manufacturer's instructions. They were then homogenized using a pellet micro‐pestle and subjected to sonication (3 cycles, 10 s each). The lysates were centrifuged at 13 000 rpm for 30 min at 4°C and the pellet discarded (Western blot analysis excluded the presence of residual DASPO, DAAO and SR proteins in the pellets). All the enzymatic assays were performed on 96‐well plates and fluorescence or absorbance signals were recorded using a microplate reader (Tecan, Infinite M Plex.).

An assay based on Amplex UltraRed has been used to measure H_2_O_2_ produced from DAAO and DASPO reactions (Rosini et al. 2017). Five–twenty microliter of the different tissue extracts were used for the assay: the solution contained 20 μM Amplex UltraRed (Thermo Fisher Scientific), 0.05 units/mL horseradish peroxidase, 2.5 mM NaN_3_, 40 μM FAD, and 100 mM D‐Ala for DAAO and 5 μM FAD and 80 mM D‐Asp for DASPO. The fluorescence of the oxidized reagent produced by DAAO or DASPO activity was recorded at 30, 60, 120, 240, 360 min, and overnight at 25°C (excitation wavelength 535 nm; emission wavelength 590 nm). A calibration curve was generated by adding known amounts of H_2_O_2_ (0.1–10 μM range). Controls with recombinant enzymes (0.2 mU for DAAO and 0.04 mU for DASPO) (Molla et al. 2006, 2020), without tissue extract samples and without substrates, were also assayed simultaneously. Moreover, as a further control, 1 mM CBIO (a DAAO inhibitor) or 20 mM meso‐tartaric acid (a DASPO inhibitor) were added to all samples. All controls were set up simultaneously to verify the specificity of the observed activity signal.

Activity assays for the β‐elimination reaction by SR were carried out with 5–20 μL of tissue extracts in an assay solution containing 50 mM triethanolamine (TEA), 500 μM L‐Ser, 2 mM ATP, 50 μM PLP, 5 mM DTT, 1 mM MgCl_2_, 150 mM NaCl, 60 U/mL lactate dehydrogenase (LDH) and 300 μM NADH, pH 8.0 (Marchetti et al. 2013). Controls with 0.5 μM of recombinant SR, without substrate or without LDH, were also set up simultaneously. A calibration curve was obtained by adding known amounts of NADH (1–300 μM range). The reaction was carried out at 25°C following the decrease of absorbance intensity at 340 nm over time (see above).

The values reported, corresponding to the ones recorded at the time of maximal activity, have been calculated by subtracting the activity value obtained by adding the specific inhibitor to the reaction solution for DASPO and DAAO, and the value obtained without adding LDH for SR. Enzymatic activity is expressed as μU normalized on μg of proteins used for the assay. One unit corresponds to the amount of enzyme that catalyzes the conversion of one μmol of substrate in 1 min. Recombinant human SR was a generous gift of Barbara Campanini's group, University of Parma, Italy.

In both HPLC detection and enzymatic activity assays, the experimenter was blinded to the group assignments, which were handled by a separate individual who also conducted the data analysis.

### 2.4 Statistical Analysis

Statistical analysis was performed using GraphPad Prism 9.0 software. A two‐way ANOVA (treatment × age) was used to evaluate differences in amino acid content or enzymatic activity between LPS‐ or VPA‐treated rats and their respective saline‐treated controls during age (adolescence and young adulthood), followed by Fisher's post hoc analysis. Unpaired Student's *t*‐test was used to evaluate differences in amino acid content or enzymatic activity between *Fmr1‐^Δ^exon 8* and WT rats. Normality distribution was tested using the Kolmogorov–Smirnov and Shapiro–Wilk tests. No test for outliers was conducted. A *p* value of less than 0.05 was considered statistically significant.

## 3 Results

### 3.1 Prenatal LPS or VPA Prenatal Treatment Does Not Affect Amino Acid Levels in the Plasma and Feces of the Rat Offspring

In this work, we used HPLC to detect plasma, fecal, and cerebral levels of D‐Asp, D‐Ser, and the main amino acids involved in glutamatergic transmission, including L‐glutamate (L‐Glu), L‐aspartate (L‐Asp), glycine (Gly), and their immediate precursors, L‐glutamine (L‐Gln), L‐asparagine (L‐Asn) and L‐serine (L‐Ser) (Figure 1).

In the plasma of LPS‐exposed rats, two‐way ANOVA analysis revealed non‐significant changes in any amino acid detected during life compared with saline‐exposed controls (Table S1). We found very low levels of D‐Asp at P35, which fell below the detection limit of our HPLC settings (0.01 pmol) at P75 (Table S1). Similarly, in VPA‐exposed rats, all amino acid levels were comparable between treatments in the time window analyzed (Table S2). In this rat model, D‐Asp levels were below the detection limit at both P40 and P90 (Table S2).

We also examined whether the levels of D‐Asp, D‐Ser, and other amino acids were altered in the feces of LPS‐ or VPA‐exposed rats. However, similar to the plasma results, no significant changes in the amino acid levels were detected in either environmental ASD rat model at any age examined (Tables S3 and S4).

Overall, no amino acid level alterations were found in the plasma and feces of both rat models of ASD.

### 3.2 Prenatal Exposure to LPS Increases D‐Asp Levels in the Prefrontal Cortex of Adolescent Rats

**FIGURE 2:**
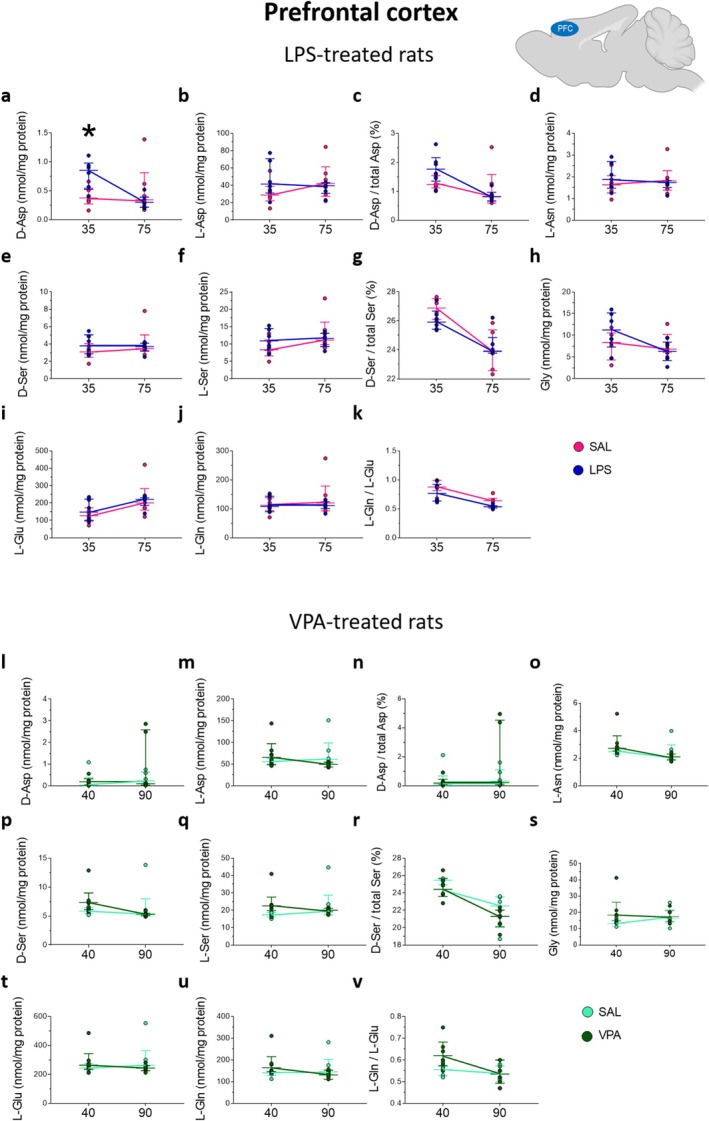
Amino acid levels in the prefrontal cortex of rats prenatally treated with LPS or VPA at adolescence and young adulthood. Analysis of (a, l) D‐aspartate and (b, m) L‐aspartate levels, (c, n) D‐aspartate/total aspartate ratio, (d, o) L‐asparagine, (e, p) D‐serine and (f, q) L‐serine levels, (g, r) D‐serine/total serine ratio, (h, s) glycine, (i, t) L‐glutamate (j, u) and L‐glutamine levels, (k, v) L‐glutamine/L‐glutamate ratio in the prefrontal cortex of (a–k) LPS‐ or (l–v) VPA‐treated rats and their relative saline‐treated controls (*n* = 6 samples/treatment/age). The amino acid content was expressed as nmol/mg protein, while the ratios were expressed as % (D‐aspartate/total aspartate and D‐serine/total serine) or absolute values (L‐glutamine/L‐glutamate). In each sample, free amino acids were detected in a single run. *p < 0.05, compared with age‐matched saline‐treated rats (Fisher's post hoc comparison). Dots represent the single subject's values while bars illustrate the median with interquartile range.

ANOVA analysis in the PFC revealed that D‐Asp levels changed significantly between LPS‐ and saline‐exposed rats over time (treatment × age: *F*_(1,20)_ = 6.579, *p* = 0.0185). The following Fisher's post hoc analysis evidenced a selective increase in D‐Asp levels in LPS‐exposed rats at P35, compared with age‐matched rats prenatally exposed to saline (median [IQR] of nmol/mg protein: saline = 0.37 [0.27; 0.55] vs. LPS = 0.85 [0.40; 0.98]; *p* = 0.0267; Figure 2a). No time‐dependent variations in the other detected amino acids were found (Figure 2b–j). In rats exposed prenatally to VPA, we found an age‐dependent effect of this stressor on L‐Gln levels (*F*_(1,20)_ = 5.184, *p* = 0.0339), which, however, did not result in significant variations at each age analyzed.

Overall, in the PFC, we found a selective increase in D‐Asp levels only in adolescent LPS‐exposed rats, compared to the respective control rats.

### 3.3 Prenatal Exposure to LPS Increases D‐Asp and Gly Levels in the Hippocampus of Adolescent Rats

**FIGURE 3:**
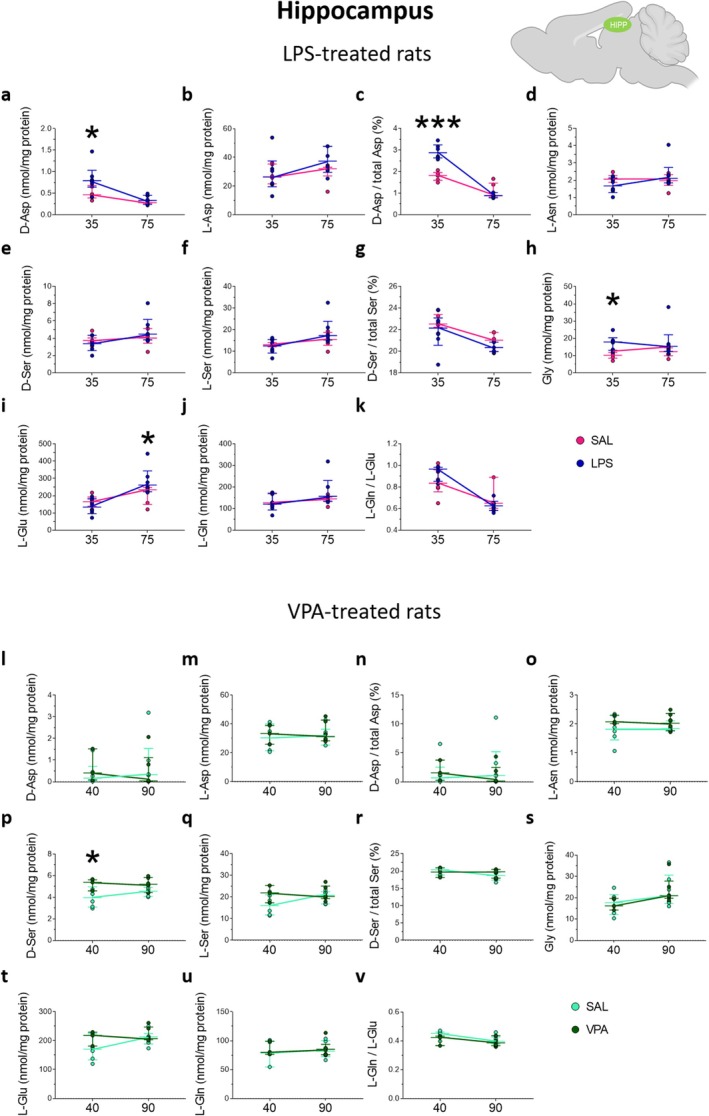
Amino acid levels in the hippocampus of rats prenatally treated with LPS or VPA at adolescence and young adulthood. Analysis of (a, l) D‐aspartate and (b, m) L‐aspartate levels, (c, n) D‐aspartate/total aspartate ratio, (d, o) L‐asparagine, (e, p) D‐serine and (f, q) L‐serine levels, (g, r) D‐serine/total serine ratio, (h, s) glycine, (i, t) L‐glutamate (j, u) and L‐glutamine levels, (k, v) L‐glutamine/L‐glutamate ratio in the hippocampus of (a–k) LPS‐ or (l–v) VPA‐treated rats and their relative saline‐treated controls (*n* = 6 samples/treatment/age except for VPA‐exposed rats at P40, *n* = 3 samples). The amino acid content was expressed as nmol/mg protein, while the ratios were expressed as % (D‐aspartate/total aspartate and D‐serine/total serine) or absolute values (L‐glutamine/L‐glutamate). In each sample, free amino acids were detected in a single run. *p < 0.05, ***p < 0.0001, compared with age‐matched saline‐treated rats (Fisher's post hoc comparison). Dots represent the single subject's values while bars illustrate the median with interquartile range.

Statistical analysis revealed that prenatal LPS exposure significantly affected D‐Asp levels in the hippocampus (*F*_(1,20)_ = 5.011, *p* < 0.0367; Figure 3a) and produced an age‐dependent variation in D‐Asp/total Asp ratio in rat offsprings (treatment × age: *F*_(1,20)_ = 34.20, *p* < 0.0001; treatment: *F*_(1,20)_ = 148.2, *p* < 0.0001). In particular, post hoc analysis showed that D‐Asp levels and D‐Asp/total Asp ratio were higher in LPS‐exposed rats selectively at P35, compared to animals of the same age prenatally exposed to saline (D‐Asp: saline: 0.46 [0.39; 0.68] vs. LPS = 0.79 [0.64; 1.03]; *p* = 0.0105; D‐Asp/total Asp: saline = 1.82 [1.60; 1.96] vs. LPS = 2.88 [2.64; 3.24]; *p* < 0.0001; Figure 3c). In addition, we revealed a significant main effect of LPS treatment on Gly levels (*F*_(1,20)_ = 8.425, *p* = 0.0088), which evidenced a selective Gly increase in LPS‐exposed rats at P35, compared to saline‐exposed controls (saline = 10.19 [8.46; 11.76] nmol/mg protein vs. LPS = 17.89 [13.10; 20.37] nmol/mg protein; *p* = 0.0376; Figure 3h). Finally, ANOVA analysis showed a significant age‐dependent effect of prenatal LPS treatment on L‐Glu levels (*F*_(1,20)_ = 5.286, *p* = 0.0324), revealing an increase of this amino acid levels in P75 rats, compared to age‐matched rats prenatally exposed to saline (saline = 234.6 [149.6; 248.3] vs. LPS = 262.1 [223.4; 343.6]; *p* = 0.0291; Figure 3i).

On the other hand, prenatal VPA exposure only affected D‐Ser levels (*F*_(1,17)_ = 7.036, *p* = 0.0168), producing a significant increase in P40 VPA‐exposed rats, compared to age‐matched rats prenatally exposed to saline (saline = 3.96 [3.09; 4.97] vs. VPA = 5.39 [4.60; 5.62]; *p* = 0.0432; Figure 3p).

Altogether, in the hippocampus, we observed an increase in D‐Asp and Gly levels in adolescent LPS‐exposed rats compared to control animals. The increase in D‐Asp led to a higher D‐Asp/total Asp ratio in LPS‐exposed rats. In young adult rats prenatally exposed to LPS, we found elevated L‐Glu levels compared with age‐matched control rats. In contrast, in VPA‐exposed rats, our analysis revealed a statistically significant increase only in D‐Ser levels at the adolescent phase, compared with saline‐exposed rats.

### 3.4 Prenatal Exposure to LPS or VPA Increases D‐Asp Levels in the Dorsal Striatum of Adolescent Rats

**FIGURE 4:**
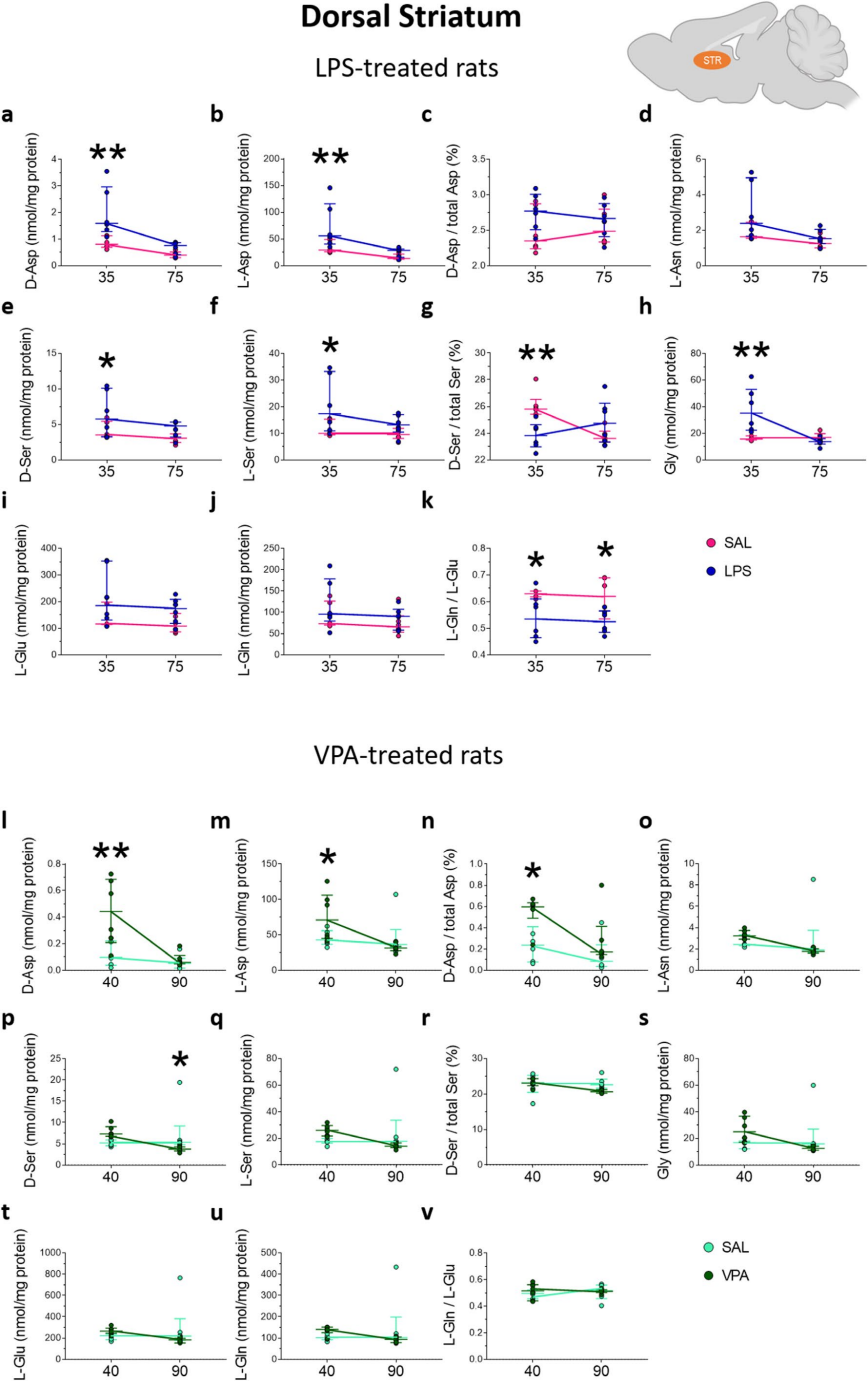
Amino acid levels in the dorsal striatum of rats prenatally treated with LPS or VPA at adolescence and young adulthood. Analysis of (a, l) D‐aspartate and (b, m) L‐aspartate levels, (c, n) D‐aspartate/total aspartate ratio, (d, o) L‐asparagine, (e, p) D‐serine and (f, q) L‐serine levels, (g, r) D‐serine/total serine ratio, (h, s) glycine, (i, t) L‐glutamate (j, u) and L‐glutamine levels, (k, v) L‐glutamine/L‐glutamate ratio in the dorsal striatum of (a–k) LPS‐ or (l–v) VPA‐treated rats and their relative saline‐treated controls (*n* = 6 samples/treatment/age). The amino acid content was expressed as nmol/mg protein, while the ratios were expressed as % (D‐aspartate/total aspartate and D‐serine/total serine) or absolute values (L‐glutamine/L‐glutamate). In each sample, free amino acids were detected in a single run. *p < 0.05, **p < 0.01, compared with age‐matched saline‐treated rats (Fisher's post hoc comparison). Dots represent the single subject's values while bars illustrate the median with interquartile range.

We found a significant main effect of LPS treatment on D‐Asp and L‐Asp levels in the dorsal striatum (D‐Asp: *F*_(1,20)_ = 11.01, *p* = 0.0034; L‐Asp: *F*_(1,20)_ = 6.436, *p* = 0.0196; Figure 4a,b). The following post hoc analyses evidenced a substantial increase for both Asp enantiomers at P35 (D‐Asp: saline = 0.80 [0.69; 1.13] nmol/mg protein vs. LPS = 1.59 [1.28; 2.96] nmol/mg protein; *p* = 0.0011; L‐Asp: saline = 29.18 [25.87; 48.97] nmol/mg protein vs. LPS = 55.87 [40.45; 115.8] nmol/mg protein; *p* = 0.0085; Figure 4a,b). Similarly, D‐Ser and L‐Ser levels significantly changed in response to prenatal LPS administration (treatment: D‐Ser: *F*_(1,20)_ = 5.374, *p* = 0.0311; L‐Ser: *F*_(1,20)_ = 6.088, *p* = 0.0228; Figure 4e,f), which induced a significant increase of both amino acids at P35 (D‐Ser: saline = 3.51 [3.23; 5.45] nmol/mg protein vs. LPS = 5.75 [3.26; 10.09] nmol/mg protein; *p* = 0.0478; L‐Ser: saline = 10.03 [9.45; 15.33] nmol/mg protein vs. LPS = 17.38 [10.96; 33.29] nmol/mg protein; *p* = 0.0190; Figure 4e,f). As a consequence of D‐Ser and L‐Ser variations, a significant influence of prenatal LPS treatment on D‐Ser/total Ser ratio over time was apparent (*F*_(1,20)_ = 13.00, *p* = 0.0018; Figure 4g), resulting in a selective decrease of this parameter at P35 (saline = 25.81 [25.42; 26.54] % vs. LPS = 23.83 [22.97; 24.64] %; *p* = 0.0029; Figure 4g). The prenatal LPS treatment also affected Gly levels during life (*F*_(1,20)_ = 12.10, *p* = 0.0024; Figure 4h), which resulted in a significant increase in LPS‐exposed rats at P35, compared to saline‐exposed animals (saline = 15.72 [15.14; 18.08] vs. LPS = 35.26 [22.50; 53.11]; *p* = 0.0004; Figure 4h). Finally, ANOVA analysis evidenced a main effect of prenatal LPS treatment on L‐Gln/L‐Glu ratio in the offspring (*F*_(1,20)_ = 10.87, *p* = 0.0036; Figure 4h), resulting in a reduced L‐Gln/L‐Glu ratio at both P35 and P75 (P35: saline = 0.63 [0.62; 0.64] vs. LPS = 0.53 [0.46; 0.61]; *p* = 0.0289; P75: saline = 0.62 [0.53; 0.69] vs. LPS = 0.52 [0.48; 0.56]; *p* = 0.0318).

In the dorsal striatum of rats prenatally exposed to VPA, we found that D‐Asp and L‐Asp levels were affected in an age‐dependent manner (D‐Asp: treatment × age: *F*_(1,20)_ = 6.927, *p* = 0.0160; treatment: *F*_(1,20)_ = 8.897, *p* = 0.0074; L‐Asp: treatment × age: *F*_(1,20)_ = 5.750, *p* = 0.0264; Figure 4l,m). The following post hoc analysis revealed increased levels of both Asp enantiomers in VPA‐exposed rats at P40, compared to age‐matched rats exposed to saline (D‐Asp: saline = 0.10 [0.02; 0.22] vs. VPA = 0.44 [0.21; 0.69]; *p* = 0.0008; L‐Asp: saline = 42.77 [36.74; 55.95] vs. VPA = 70.93 [44.98; 106.1]; *p* = 0.0378; Figure 4l,m). As a consequence of Asp enantiomer changes, prenatal VPA treatment also modified the D‐Asp/total Asp ratio in the offspring (treatment: *F*_(1,20)_ = 7.157, *p* = 0.0145), leading to an increase selectively at P40, compared to saline treatment (saline = 0.24 [0.08; 0.41] vs. VPA = 0.60 [0.49; 0.63]; *p* = 0.0199; Figure 4n).

We also found that prenatal VPA treatment altered the levels of D‐Ser, L‐Ser, and the L‐Ser derivative, Gly, over time (D‐Ser: *F*_(1,20)_ = 6.664, *p* = 0.0178; L‐Ser: *F*_(1,20)_ = 4.456, *p* = 0.0476; Gly: *F*_(1,20)_ = 5.594, *p* = 0.0282; Figure 4p,q,s). Such changes were reflected in a significant decrease in D‐Ser levels, but not in L‐Ser and Gly alterations, in VPA‐exposed rats at P90 relative to age‐matched control rats (saline = 5.35 [4.81; 9.23] vs. VPA = 3.76 [3.31; 4.28]; *p* = 0.0437; Figure 4p).

Overall, in the dorsal striatum, we observed a significant increase in D‐Asp, L‐Asp, D‐Ser, L‐Ser, and Gly levels, along with a decrease of both D‐Ser/total Ser and L‐Gln/L‐Glu ratios in adolescent LPS‐exposed rats compared to saline‐exposed animals. A reduced L‐Gln/L‐Glu ratio was also observed in LPS‐exposed rats in young adulthood. In VPA‐exposed rats, we reported increased D‐Asp, L‐Asp, and D‐Asp/total Asp ratios in adolescence, as well as elevated D‐Ser levels compared to their respective age‐matched controls.

### 3.5 Prenatal Exposure to VPA Increases D‐Asp, D‐Ser, and Other Amino Acid Levels in the Nucleus Accumbens of Adolescent Rats

**FIGURE 5:**
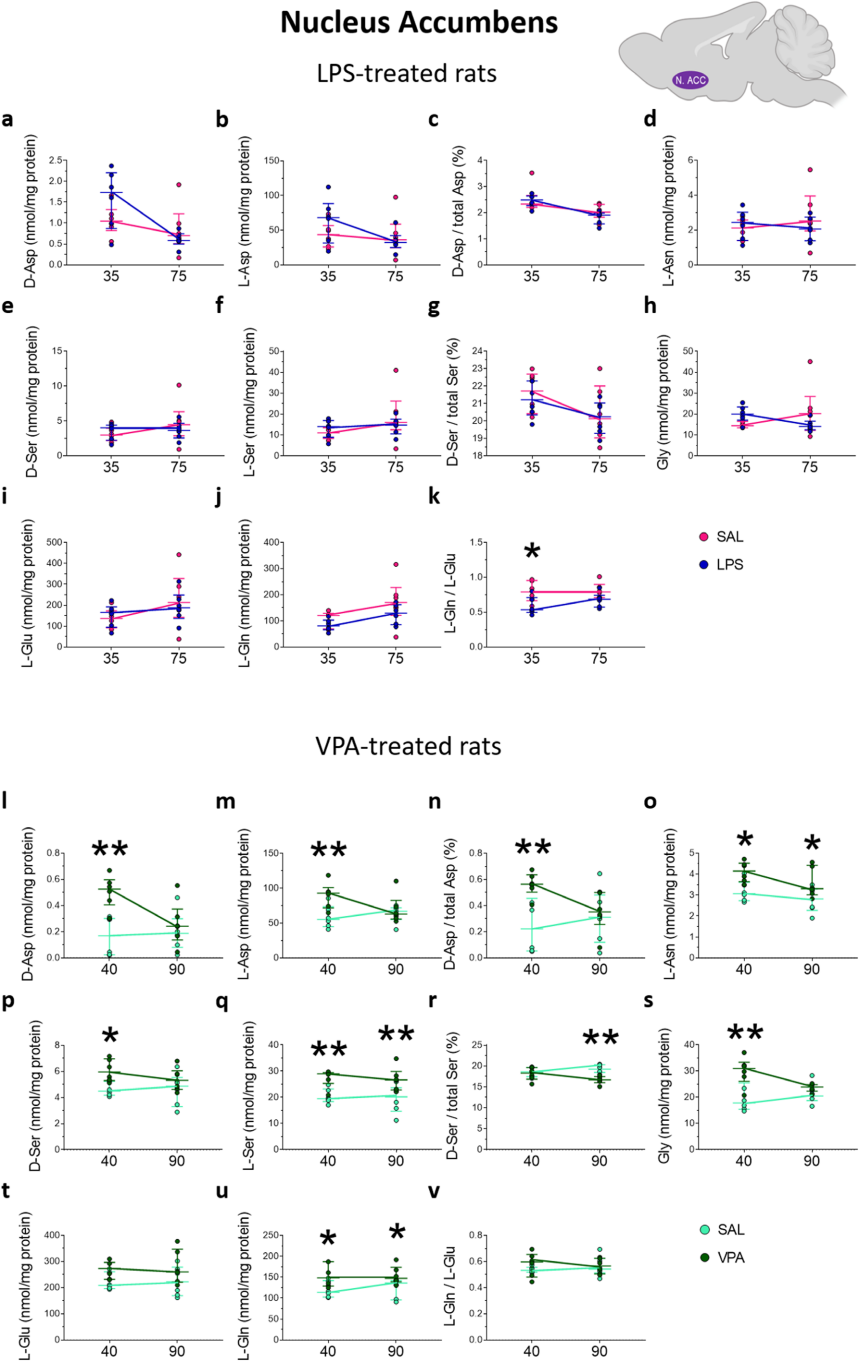
Amino acid levels in the nucleus accumbens of rats prenatally treated with LPS or VPA at adolescence and young adulthood. Analysis of (a, l) D‐aspartate and (b, m) L‐aspartate levels, (c, n) D‐aspartate/total aspartate ratio, (d, o) L‐asparagine, (e, p) D‐serine and (f, q) L‐serine levels, (g, r) D‐serine/total serine ratio, (h, s) glycine, (i, t) L‐glutamate (j, u) and L‐glutamine levels, (k, v) L‐glutamine/L‐glutamate ratio in the nucleus accumbens of (a–k) LPS‐ or (l–v) VPA‐treated rats and their relative saline‐treated controls (*n* = 6 samples/treatment/age). The amino acid content was expressed as nmol/mg protein, while the ratios were expressed as % (D‐aspartate/total aspartate and D‐serine/total serine) or absolute values (L‐glutamine/L‐glutamate). In each sample, free amino acids were detected in a single run. *p < 0.05, **p < 0.01, compared with age‐matched saline‐treated rats (Fisher's post hoc comparison). Dots represent the single subject's values while bars illustrate the median with interquartile range.

In the nucleus accumbens of rats prenatally exposed to LPS, we found significant age‐dependent changes in Gly levels between treatments (*F*_(1,20)_ = 5.059, *p* = 0.0359), although no differences were found at each single time point with Fisher's post hoc comparison (Figure 5h). Moreover, ANOVA analysis evidenced a significant main effect of prenatal LPS treatment on L‐Gln/L‐Glu ratio in the offspring (*F*_(1,20)_ = 10.95, *p* = 0.0035), which resulted in a significant reduction in this parameter at P35 (saline = 0.79 [0.71; 0.90] vs. LPS = 0.68 [0.57; 0.74]; *p* = 0.0093; Figure 5k).

In VPA‐exposed rats, a significant age‐dependent variation in D‐Asp levels between treatments and a main effect of prenatal VPA treatment on L‐Asp and D‐Asp/total Asp ratio was observed (treatment × age: D‐Asp: *F*_(1,20)_ = 5.276, *p* = 0.0325; treatment: L‐Asp: *F*_(1,20)_ = 6.929, *p* = 0.0160; D‐Asp/total Asp: *F*_(1,20)_ = 5.562, *p* = 0.0286; Figure 5l–n). Such variations resulted in increased D‐Asp and L‐Asp levels and D‐Asp/total Asp ratio in P40 rats prenatally exposed to VPA compared to saline‐exposed controls (D‐Asp: saline = 0.17 [0.02; 0.30] vs. VPA = 0.53 [0.40; 0.60]; *p* = 0.0008; L‐Asp: saline = 55.14 [44.96; 73.34] vs. VPA = 92.99 [71.77; 100.9]; *p* = 0.0058; D‐Asp/total Asp: saline = 0.22 [0.05; 0.46] vs. VPA = 0.56 [0.50; 0.64]; *p* = 0.0074; Figure 5l–n). We also found an effect of VPA treatment on L‐Asn levels (*F*_(1,20)_ = 12.85, *p* = 0.0019), which evidenced an increase in rats exposed to VPA at both P40 and P90 compared with the respective age‐matched control rats (P40: saline = 3.07 [2.73; 3.62] vs. VPA = 4.15 [3.64; 4.52]; *p* = 0.0130; P90: saline = 2.82 [2.27; 3.30] vs. VPA = 3.30 [3.02; 4.42]; *p* = 0.0296; Figure 5o).

Statistical analysis also showed a main effect of prenatal VPA administration on offspring D‐Ser and L‐Ser levels (D‐Ser: *F*_(1,20)_ = 8.575, *p* = 0.0083; L‐Ser: *F*_(1,20)_ = 18.61, *p* = 0.0003; Figure 5p,q), and an age‐dependent effect of VPA on offspring D‐Ser/total Ser ratio (*F*_(1,20)_ = 5.332, *p* = 0.0317; Figure 5r). Post hoc analysis revealed increased levels of D‐Ser at P40 and L‐Ser at both P40 and P90, and decreased D‐Ser/total Ser ratio at P90 in VPA‐exposed rats, compared to the respective age‐matched saline‐exposed animals (D‐Ser: P40: saline = 4.50 [4.17; 5.23] vs. VPA = 5.96 [5.30; 6.96]; *p* = 0.0136; L‐Ser: P40: saline = 19.53 [18.38; 23.22] vs. VPA = 29.03 [25.30; 29.44]; *p* = 0.0079; P90: saline = 20.19 [14.67; 23.67] vs. VPA = 26.64 [22.86; 29.89]; *p* = 0.0050; D‐Ser/total Ser ratio: P90: saline = 19.21 [18.50; 20.30] % vs. VPA = 16.71 [16.03; 17.47]; *p* = 0.0009; Figure 5p–r). Moreover, we found a main effect of prenatal VPA treatment on Gly levels (*F*_(1,20)_ = 9.776, *p* = 0.0053; Figure 5s), which resulted in a significantly higher content of this amino acid in VPA‐exposed rats selectively at P40 (saline = 17.82 [15.40; 25.50] vs. VPA = 30.94 [26.10; 33.43]; *p* = 0.0019; Figure 5s). L‐Ser levels in the brain are known to be partially modulated by endogenous synthesis due to the phosphorylated pathway (Murtas et al. 2020): the level of L‐Ser affects D‐Ser ones through the activity of SR and Gly levels through serine hydroxymethyltransferase. Finally, statistical analysis revealed a main effect of VPA exposure on offspring L‐Glu and L‐Gln levels (L‐Glu: *F*_(1,20)_ = 5.827, *p* = 0.0255; L‐Gln: *F*_(1,20)_ = 11.06, *p* = 0.0034; Figure 5t,u). Post hoc analysis showed significant changes only in L‐Gln levels that were increased in VPA‐exposed rats at both P40 and P90, compared to the respective age‐matched controls (P40: saline = 113.9 [102.6; 142.2] vs. VPA = 148.4 [128.8; 187.3]; *p* = 0.0208; P90: saline = 136.1 [95.69; 142.5] vs. VPA = 147.3 [139.3; 173.6]; *p* = 0.0403; Figure 5u).

Altogether, in LPS‐exposed rats, we observed a significant decrease only in the L‐Gln/L‐Glu ratio in the adolescent offspring, compared to saline‐exposed rats. In contrast, in VPA‐exposed rats, an increase in D‐Asp, L‐Asp, L‐Asn, D‐Ser, L‐Ser, Gly, and L‐Gln levels in adolescence compared to age‐matched controls was apparent. In young adulthood, VPA‐exposed rats showed a significant increase in L‐Asn, L‐Ser, and L‐Gln levels, as well as a decrease in the D‐Ser/total Ser ratio, compared to saline‐exposed rats.

### 3.6 Prenatal Exposure to LPS Increases D‐Asp and Gly Levels in the Amygdala of Adolescent Rats

**FIGURE 6:**
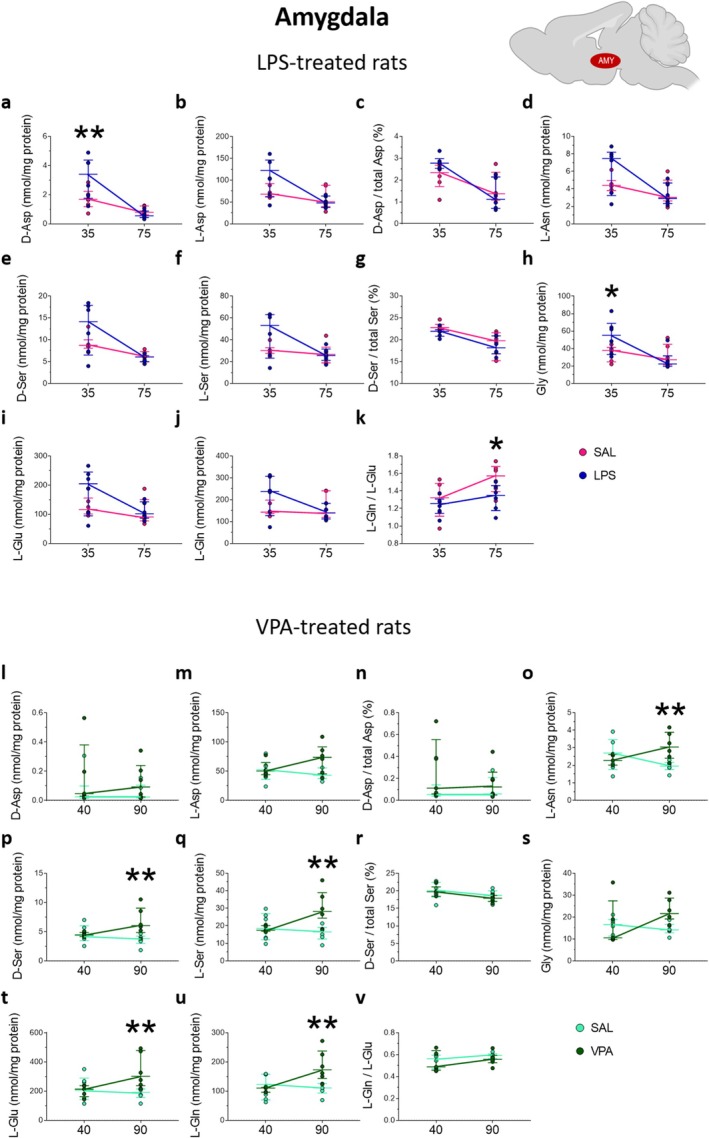
Amino acid levels in the amygdala of rats prenatally treated with LPS or VPA at adolescence and young adulthood. Analysis of (a, l) D‐aspartate and (b, m) L‐aspartate levels, (c, n) D‐aspartate/total aspartate ratio, (d, o) L‐asparagine, (e, p) D‐serine and (f, q) L‐serine levels, (g, r) D‐serine/total serine ratio, (h, s) glycine, (i, t) L‐glutamate (j, u) and L‐glutamine levels, (k, v) L‐glutamine/L‐glutamate ratio in the amygdala of (a–k) LPS‐ or (l–v) VPA‐treated rats and their relative saline‐treated controls (*n* = 6 samples/treatment/age except for VPA‐exposed rats at P40, *n* = 5 samples). The amino acid content was expressed as nmol/mg protein, while the ratios were expressed as % (D‐aspartate/total aspartate and D‐serine/total serine) or absolute values (L‐glutamine/L‐glutamate). In each sample, free amino acids were detected in a single run. *p < 0.05, **p < 0.01, compared with age‐matched saline‐treated rats (Fisher's post hoc comparison). Dots represent the single subject's values while bars illustrate the median with interquartile range.

In the amygdala of rats prenatally exposed to LPS, we found a significant age‐dependent effect on D‐Asp levels (*F*_(1,20)_ = 6.254, *p* = 0.0212), which revealed significantly higher D‐Asp content in P35 LPS‐exposed rats compared to age‐matched saline‐exposed controls (saline = 1.68 [1.18; 2.23] vs. LPS = 3.40 [1.75; 4.38]; *p* = 0.0067; Figure 6a). Similarly, statistical analysis revealed that LPS affected Gly levels over time (*F*_(1,20)_ = 4.538, *p* = 0.0458), producing an increase in these amino acid levels in LPS‐exposed rats specifically at P35 (saline = 37.89 [24.81; 41.25] vs. LPS = 55.22 [33.19; 68.88]; *p* = 0.0286; Figure 6h). We also found a main effect of prenatal LPS treatment on the L‐Gln/L‐Glu ratio (*F*_(1,20)_ = 4.430, *p* = 0.0482), resulting in a decrease of this parameter in P75 LPS‐exposed rats compared to age‐matched control animals (saline = 1.57 [1.39; 1.68] vs. LPS = 1.35 [1.18; 1.46]; *p* = 0.0311; Figure 6k).

In rats prenatally exposed to VPA, we found a significant age‐dependent effect of this drug on L‐Asn levels (*F*_(1,19)_ = 7.135, *p* = 0.0151), which disclosed an increased content of this amino acid selectively in P90 VPA‐exposed animals compared to age‐matched control rats (saline = 1.96 [1.76; 2.16] vs. VPA = 3.04 [2.40; 3.88]; *p* = 0.0077; Figure 6o). We also evidenced that prenatal VPA treatment affected D‐Ser and L‐Ser levels in an age‐dependent manner (D‐Ser: *F*_(1,19)_ = 5.871, *p* = 0.0255; L‐Ser: *F*_(1,19)_ = 7.130, *p* = 0.0151), producing an increase in both Ser enantiomers in VPA‐exposed animals at P90, compared with saline‐exposed rats of the same age (D‐Ser: saline = 3.84 [2.99; 4.24] vs. VPA = 6.01 [4.87; 9.05]; *p* = 0.0031; L‐Ser: saline = 16.52 [12.48; 19.05] vs. VPA = 28.21 [24.38; 38.98]; *p* = 0.0017; Figure 6p,q). Finally, we found an age‐dependent variation of L‐Glu and L‐Gln levels (L‐Glu: *F*_(1,19)_ = 6.122, *p* = 0.0229; L‐Gln: *F*_(1,19)_ = 7.726, *p* = 0.0119), which resulted in a significant increase of these amino acids in rats prenatally exposed to VPA, compared to those of the same age exposed to saline (L‐Glu: saline = 192.1 [156.1; 214.7] vs. VPA = 301.6 [237.3; 478.6]; *p* = 0.0038; L‐Gln: saline = 110.7 [93.33; 125.5] vs. VPA = 172.8 [143.7; 237.1]; *p* = 0.0021; Figure 6t,u).

Overall, in the amygdala, we observed an elevation in D‐Asp and Gly levels in adolescent rats prenatally exposed to LPS, compared to age‐matched controls. Additionally, we found a reduction in L‐Gln/L‐Glu ratio in young adult LPS‐exposed rats, relative to age‐matched saline controls. Conversely, in rats prenatally exposed to VPA, we observed amino acid alterations exclusively in adulthood, with increases in L‐Asn, D‐Ser, L‐Ser, L‐Glu, and L‐Gln levels, compared with saline‐exposed animals.

### 3.7 DASPO, DAAO and SR Activity Levels in the Prefrontal Cortex and Dorsal Striatum of LPS‐ and VPA‐Exposed Rats

We analyzed the enzymatic activity of DASPO, DAAO, and SR using highly sensitive assays (to detect the activity on the low amounts of available tissues) and focusing on the PFC and dorsal striatum of LPS‐ and VPA‐exposed rats and their respective saline‐exposed controls since, especially the latter brain region, exhibited alterations in both D‐Asp and D‐Ser levels in environmental ASD models. All the activity values have been corrected for background aspecific signals, using selective enzyme inhibitors for DASPO and DAAO or removing the coupling enzyme LDH for SR. For all three enzymes, the activity was expressed as μU/μg total protein. As a general rule, the DAAO activity values were in all cases very low, while DASPO and SR levels were at least 5‐ and 100‐fold higher, respectively.

In the PFC, a statistically significant decrease in DASPO activity at P75 as compared to P35 in LPS‐exposed rats was observed, while no change was apparent in control rats (Table S5). A statistically significant increase was observed for DAAO activity in control rats at P75 as compared to P35 (as well as in LPS‐treated ones, even if the change did not reach a statistical threshold, Table S5). No statistically significant changes in SR activity level were apparent (Table S5). In the dorsal striatum, DASPO and SR activity levels showed a similar moderate increase with age in both LPS‐exposed and control samples (Table S5), while a strong and statistically significant time‐dependent DAAO increase (> 4‐fold, Table S5) was apparent for both control and LPS‐exposed animals.

In rats prenatally exposed to VPA, we found identical DASPO activity levels in the PFC and dorsal striatum compared to control animals, unchanged with age (Table S6). DAAO activity levels decreased with age in the PFC and striatum of both VPA‐exposed and control rats (Table S6). SR activity showed a time‐dependent decrease in all samples and a statistically significant lower level in PFC at P40 compared to control (Table S6).

By using the specific activity of pure rat DASPO (51.7 U/mg protein) (Katane et al. 2018), rat DAAO (40.5 U/mg protein) (Frattini et al. 2011), and mouse SR (2.2 U/mg) (Hoffman et al. 2009), the activity values were converted in protein levels (Tables S5 and S6). DAAO amount was lower than the sensitivity of Western blot analyses: 0.07–0.4 ng/mg protein estimated from the activity assay versus a lower limit of Western blot detection corresponding to ~10 ng/mg protein. DASPO level was in the 0.9–2.1 ng/mg protein range from the activity assay versus ≤ 8 ng/mg protein determined from Western blot. This is a good correspondence since the specific activity value used for the estimation of DASPO level was determined at 37°C while our activity assay was performed at 25°C, likely resulting in an underestimation of the enzyme quantity. On the contrary, the calculated amount of SR based on enzymatic activity was approx. 10‐fold higher than the value determined from Western blot analysis (0.4–1.0 μg/mg protein vs. 0.03–0.06 μg/mg protein): this result suggests a modulation of SR activity in these brain tissues.

Overall, our findings indicate that DASPO activity levels are not significantly affected by prenatal treatments or the age of the animals. In contrast, DAAO activity levels exhibit age‐dependent changes in both control and treated groups. However, given the very low activity levels observed, these changes should be interpreted with caution, as they do not appear sufficient to modulate D‐Ser levels. Finally, SR activity is high in both the PFC and striatum, and probably positively stimulated by the cellular conditions as compared to the activity determined for this PLP‐dependent enzyme under in vitro conditions, suggesting a role in regulating D‐Ser levels, which are also influenced by L‐Ser, Gly, and related metabolic pathways.

### 3.8 Deletion of *Fmr1* Gene Does Not Affect Amino Acid Levels in Plasma and Brain Regions of Adolescent Rats

**FIGURE 7:**
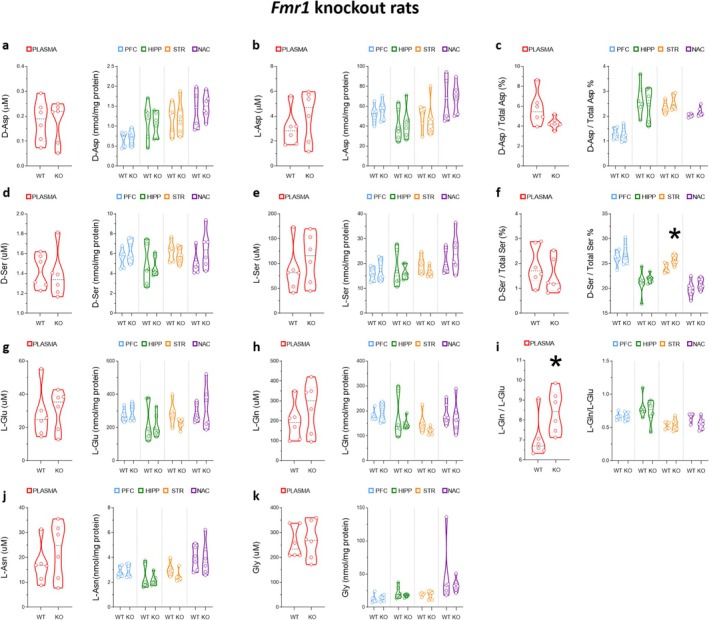
Amino acid levels in the plasma and brain regions of Fmr1‐^Δ^exon 8 knockout rats at adolescence. Analysis of (a) D‐aspartate and (b) L‐aspartate levels, (c) D‐aspartate/total aspartate ratio, (d) D‐serine and (e) L‐serine levels, (f) D‐serine/total serine ratio, (g) L‐glutamate, (h) and L‐glutamine levels, (i) L‐glutamine/L‐glutamate ratio, (j) L‐asparagine, (k) glycine levels in the plasma (left panel) and brain regions, including prefrontal cortex, hippocampus, dorsal striatum and nucleus accumbens of *Fmr1* knockout (KO) and wild‐type (WT) rats (*n* = 6 samples/genotype except for dorsal striatum, *n* = 5 WT samples). The amino acid content was expressed as μM (plasma) or nmol/mg protein (brain regions), while the ratios were expressed as % (D‐aspartate/total aspartate and D‐serine/total serine) or absolute values (L‐glutamine/L‐glutamate). In each sample, free amino acids were detected in a single run. *p < 0.05, compared with WT rats (Student's *t* test). Dots represent the single subject's values, while dotted lines illustrate the median with the interquartile range.

Finally, we measured amino acid levels in both plasma and brain regions, including the PFC, hippocampus, dorsal striatum, and nucleus accumbens, of *Fmr1‐^Δ^exon 8* rats and their WT littermates at the adolescent stage. Unpaired Student's *t*‐test analysis in the plasma revealed only a selective deregulation of the L‐Gln/L‐Glu ratio that resulted in being higher in *Fmr1‐^Δ^exon 8* rats compared with WT animals (WT = 6.70 [6.44; 7.57] vs. *Fmr1‐^Δ^exon 8* = 8.43 [7.39; 9.36]; *p* = 0.0485; Figure 7i). In brain regions, we did not reveal significant deregulations except in the dorsal striatum, where we found a significant increase in the D‐Ser/total Ser ratio in *Fmr1‐^Δ^exon 8* rats compared to WT animals (WT = 23.68 [23.29; 24.91] vs. *Fmr1‐^Δ^exon 8* = 25.72 [25.03; 26.73]; *p* = 0.0132; Figure 7f).

Altogether, in *Fmr1‐^Δ^exon 8* rats, we only found altered L‐Gln/L‐Glu and D‐Ser/total Ser ratios in the plasma and dorsal striatum, respectively, compared to WT controls.

Concerning the enzyme activity, in the PFC and dorsal striatum no significant changes in SR, DASPO, and DAAO figures have been observed between *Fmr1‐^Δ^exon 8* rats and controls, while DAAO was increased in the hippocampus and nucleus accumbens, reaching a statistically significant threshold in the former tissue only (Table S7). Furthermore, DASPO levels are 2‐ to 3‐fold lower in striatum and nucleus accumbens compared to PFC and hippocampus, and this aligns well with higher D‐Asp levels (Table S7). Compared to rats treated with VPA and LPS, the levels of DAAO, DASPO, and SR (based on activity values) in PFC and striatum were in all cases lower in *Fmr1‐^Δ^exon 8* rats: DAAO: 0.03–0.09 versus 0.07–0.4 ng/mg protein; DASPO: 0.2–0.8 versus 0.9–2.1 ng/mg protein; SR: 0.21–0.29 versus 0.4–1.0 μg/mg protein.

## 4 Discussion

In the present study, we performed a comprehensive characterization of D‐Asp, D‐Ser, and other excitatory amino acid levels in well‐established animal models of ASD, including rats prenatally exposed to the environmental stressors LPS and VPA, as well as the genetic *Fmr1‐^Δ^exon 8* rat model.

Our results revealed no significant changes in the levels of D‐Asp, D‐Ser, or other amino acids in the blood serum and feces of both environmental and genetic ASD rat models, compared to respective controls. This preclinical finding is consistent with our recent study, which reported no alterations in the levels of these amino acids in the blood of ASD patients from two Italian hospitals, compared to nonpsychiatric individuals (Garofalo et al. 2024).

Despite the lack of significant changes in peripheral amino acid levels, we observed remarkable dysregulation in neuroactive amino acid profiles across all brain regions in both LPS‐ and VPA‐exposed rats. Among the amino acids analyzed, D‐Asp exhibited the most striking dysregulation, resulting in a marked increase specifically during adolescence in several brain regions of both LPS‐ and VPA‐exposed rats. Specifically, we found significantly increased D‐Asp levels in the PFC, hippocampus, dorsal striatum, and amygdala of LPS‐exposed rats, and in the dorsal striatum and nucleus accumbens of rats prenatally exposed to VPA, compared to respective control groups. Noteworthy, in both ASD rat models, no alterations in brain D‐Asp levels were observed during adulthood. Given the physiological transient abundance of D‐Asp during prenatal and early postnatal brain development (Hashimoto et al. 1993; Wolosker et al. 2000; Sakai et al. 1998; Punzo et al. 2016), the selective early accumulation of this D‐amino acid observed during the adolescent phase suggests a delayed decline in D‐Asp levels, leading to its abnormal persistence beyond early postnatal stages in the environmental ASD models analyzed.

Although in fewer brain regions than D‐Asp, we also observed increased levels of both major NMDAR co‐agonists, D‐Ser and Gly, in adolescent LPS‐ and VPA‐exposed rats. In particular, D‐Ser levels were increased in the striatum of LPS‐exposed rats, and the hippocampus and nucleus accumbens of VPA‐exposed rats, while Gly levels were elevated in the dorsal striatum and amygdala of LPS‐exposed rats and the hippocampus of both ASD rat models.

Notably, the abnormal accumulation of endogenous NMDAR ligands—D‐Asp, D‐Ser, and Gly—in the adolescent phase coincides with the emergence of the most prominent ASD‐like behavioral manifestations in these animal models (Bergdolt and Dunaevsky 2019; Nicolini and Fahnestock 2018; Carbone et al. 2023; Schiavi et al. 2019; Melancia et al. 2018). However, it remains unclear whether the increased brain levels of these endogenous NMDAR agonists and co‐agonists are a causative factor contributing to glutamatergic signaling abnormalities reported in ASD, or whether they represent compensatory mechanisms aimed at counteracting NMDAR hypofunction. Indeed, both opposite glutamatergic dysfunctions—hyperactivation and hypofunction—have been implicated in ASD (Montanari et al. 2022). In keeping with this, pharmacological suppression of NMDAR functioning in rodents prenatally exposed to LPS or VPA has been shown to either worsen or improve ASD‐related deficits, respectively (Basta‐Kaim et al. 2011; Kang and Kim 2015; Mohammadi et al. 2020).

In line with an adaptive modification origin of abnormally higher D‐amino acids occurrence in adolescent LPS and VPA rat brains, previous preclinical studies have shown that early supplementation with D‐Ser or D‐Asp may help recover cognitive and/or synaptic plasticity impairments in ASD animal models, such as ddY mice exposed to the viral mimic polyriboinosinic–polyribo‐cytidilic acid (PolyI:C) and *Fmr1* knockout mice, respectively (Fujita et al. 2016; Li et al. 2023). Furthermore, in a recent study, we found that serum content of D‐Asp and D‐Ser transiently increases during the premorbid stages of psychosis progression, relative to non‐psychiatric controls or full‐blown schizophrenia patients (Rampino et al. 2024). These changes suggest that adaptive neurochemical responses to early glutamatergic transmission impairments may occur in the initial stages of the illness.

Among the brain regions affected by D‐Asp variations, it is noteworthy that the dorsal striatum stands out as the only region where increases in D‐Asp levels are observed in both environmental ASD rat models. In our analysis, no other amino acid changes are concurrently observed in the same brain region across both ASD rat models. Since the dorsal striatum is the brain region most densely innervated by midbrain dopaminergic afferents, it can be hypothesized that abnormally higher D‐Asp levels in this brain area may influence the glutamate‐dopamine neurotransmission interaction and, in turn, the physiological circuitry maturation in adolescent LPS‐ and VPA‐exposed rats. This is particularly relevant since ASD models commonly exhibit dysfunctions in dopamine signaling in the striatum, as reported by our and other studies (Schiavi et al. 2019; Squillace et al. 2014; Kirsten et al. 2012; Hara et al. 2015).

The present data on the selective deregulation of cerebral D‐Asp levels in LPS‐ and VPA‐exposed rats, but not in *Fmr1‐^Δ^exon 8* rats, are consistent with our previous findings, which showed increased D‐Asp levels in the PFC and hippocampus of the idiopathic ASD mouse model BTBR, but not in the same brain regions of mice with deletion of *Cntnap2*, *Shank3* genes, or *16p11.2* locus (Nuzzo et al. 2020). Considering the multifactorial origins of ASD, our results indicate that an abnormal accumulation of D‐Asp in the brain may represent a distinct neurochemical signature for environmental/idiopathic animal models of ASD, which is not observed in genetically modified models targeting single genes.

Despite the significant increases in striatal D‐Asp and D‐Ser levels observed in LPS‐ and VPA‐exposed rats, we found that the activity of the enzymes directly controlling their levels—that is, DASPO, DAAO, and SR—showed limited alterations in samples from ASD and control rats, mainly related to an age‐dependent effect. While it is well established that cerebral D‐Ser content is regulated by the coordinated activity of DAAO and SR, which control its biosynthesis and degradation, respectively (Pollegioni et al. 2018; Wolosker et al. 1999), the biosynthetic pathway of D‐Asp remains unclear, although SR likely appears to contribute to D‐Asp formation in certain brain areas (Horio et al. 2013; Ito et al. 2016). Notably, SR activity was approximately 10‐fold higher than expected based on Western blot analysis and the specific activity of mouse SR (no data on rat SR are available). SR activity is finely regulated by several physiological effectors (from ATP to cations/anions), interacting proteins, and post‐translational modifications (Raboni et al. 2018), whose fluctuations contribute to D‐Ser homeostasis. As a general rule, the very low levels of DAAO expression (here selectively determined assaying its activity) suggest a minor role for this flavoenzyme in regulating D‐Ser in the brain regions here tested. This finding indicates a finely tuned enzymatic network controlling D‐Ser concentration, highlighting the importance of L‐Ser production via the phosphorylated pathway and its conversion with Gly. Among the enzymes analyzed, SR exhibits the highest activity in both the PFC and striatum, suggesting a more significant role compared to DAAO. For instance, the time‐dependent decline in SR activity in the PFC correlates with the observed decrease in D‐Ser levels. DASPO is an enzyme with high specific activity and strong FAD interaction (Molla et al. 2020; Puggioni et al. 2020; Caldinelli et al. 2010). Its level is lower in the striatum and nucleus accumbens compared to the PFC and hippocampus, which aligns well with higher D‐Asp levels. However, it does not appear to be the primary regulator of D‐Asp levels in LPS‐ and VPA‐treated animals in either the PFC or striatum. Therefore, the possibility that additional biosynthetic mechanisms contribute to the D‐Asp variations observed in our study cannot be ruled out. Furthermore, it can be hypothesized that cerebral accumulation of these D‐amino acids may depend on dysfunctions in metabolically relevant peripheral organs, even though plasma levels of D‐Asp and D‐Ser show no significant changes in both LPS‐ and VPA‐exposed rats compared to controls. In this regard, an increasing body of research suggests that the gut microbiome plays a crucial role in regulating systemic levels of D‐amino acids, including D‐Asp and D‐Ser (Suzuki et al. 2022; Matsumoto et al. 2018; Sasabe et al. 2016; Gonda et al. 2023; Kawase et al. 2017). Given that microbiome alterations are frequently observed in ASD and that perturbations in the maternal gut microbiome can induce ASD‐relevant phenotypes in offspring (Vernocchi et al. 2022; Hughes et al. 2018; Morel et al. 2023), we also determined fecal amino acid content in LPS‐ and VPA‐exposed rats. However, our HPLC analysis did not reveal significant changes in D‐ or L‐amino acid levels in the feces of these environmental ASD rat models compared to controls. Nonetheless, future studies in patients are warranted to further explore the potential contribution of microbiome‐induced dysmetabolism of D‐Asp and D‐Ser in ASD.

In contrast to the adolescent phase, young‐adult LPS‐ and VPA‐exposed rats exhibit fewer amino acid variations, with region‐specific changes in L‐Glu, D‐Ser, L‐Ser, L‐Asn, or L‐Gln levels. Nevertheless, similar to the juvenile phase, all significant amino acid changes observed in this manifest symptomatic phase of the disease consistently trend towards increases when compared to the respective saline‐exposed controls. Interestingly, the observed increases in excitatory amino acids and their precursors in both juvenile and adult environmental ASD rats align with a broader pattern of glutamatergic dysfunction reported in animal models of MIA or prenatal VPA exposure (Bergdolt and Dunaevsky 2019; Nicolini and Fahnestock 2018; Montanari et al. 2022).

Importantly, as this study included only male rats, the findings may not be directly generalizable to females. Given the well‐established sex differences in preclinical models of ASD (Napolitano et al. 2022) and the distinct neurobiological mechanisms reported between males and females (Kamalmaz et al. 2023), future studies should incorporate both sexes to provide a more comprehensive understanding of these effects. Investigating the behavioral and neurochemical phenotypes of female offspring prenatally exposed to LPS or VPA, as well as *Fmr1‐^Δ^exon 8* rats, will be essential for determining whether the observed alterations are male‐specific or extend to females. This, in turn, will enhance the translational relevance and applicability of our findings. Furthermore, it is important to emphasize that this study represents the first comprehensive investigation of D‐ and L‐amino acid profiles in LPS‐ and VPA‐exposed rats. Consequently, independent replication using alternative experimental approaches and larger sample sizes will be essential to validate and reinforce the robustness of these preliminary findings, thereby supporting their relevance within the broader context of ASD research.

In conclusion, our findings highlight remarkable dysregulations in D‐Asp and D‐Ser levels in the brain of the adolescent offspring prenatally exposed to either LPS or VPA, two validated environmental models of ASD. Notably, such variations do not occur peripherally, in either the blood or feces, suggesting that the D‐amino acid changes observed in these environmental ASD rat models are likely driven by central metabolic alterations rather than gut microbiota metabolism or intestinal absorption/excretion processes. Our findings support the hypothesis that dysfunctions in glutamatergic neurotransmission at NMDARs and mGluR5 contribute significantly to the pathophysiology of ASD and suggest that epigenetic and neuroinflammatory mechanisms may underlie the dysregulation of brain D‐amino acid metabolism in these conditions. Unlike genetically modified rat models, these selective neurochemical alterations underscore the importance of prenatal environmental factors in shaping early changes in cerebral D‐Asp and D‐Ser levels associated with ASD, highlighting the need for further investigation into the underlying mechanisms and their implications for therapeutic strategies.

## Author Contributions

**Anna Di Maio:** investigation, visualization, formal analysis. **Isar Yahyavi:** investigation, visualization, formal analysis. **Valeria Buzzelli:** resources. **Zoraide Motta:** investigation, formal analysis. **Fabrizio Ascone:** resources. **Lorenza Putignani:** writing – review and editing. **Alessandro Usiello:** conceptualization, funding acquisition, supervision, writing – review and editing. **Loredano Pollegioni:** funding acquisition, supervision, writing – review and editing. **Viviana Trezza:** resources, supervision, writing – review and editing. **Francesco Errico:** funding acquisition, supervision, writing – original draft, writing – review and editing.

## Conflicts of Interest

Viviana Trezza is currently the Reviews Handling Editor of the *Journal of Neurochemistry*. All the other authors declare no conflicts of interest.

### Peer Review

The peer review history for this article is available at https://www.webofscience.com/api/gateway/wos/peer‐review/10.1111/jnc.70095.

## Supporting information


Data S1.

## Data Availability

The datasets used and analyzed in the current study are available from the corresponding authors upon reasonable request.
