# Supplementary material for: Prenatal Exposure to Lipopolysaccharide or Valproate Leads to Abnormal Accumulation of the NMDA Receptor Agonist D‐Aspartate in the Adolescent Rat Brain
Source: J Neurochem. 2025 May 28;169(6):e70095. doi: 10.1111/jnc.70095 (PMC12120390; doi:10.1111/jnc.70095)
Supplement: Supplementary file 1 — Data S1. [file JNC-169-0-s001.pdf]

Supplementary information for

Prenatal exposure to lipopolysaccharide or valproate leads to abnormal accumulation of the NMDA receptor agonist D-aspartate in the adolescent rat brain

Anna Di Maio[1,2,†], Isar Yahyavi[1,2,†], Valeria Buzzelli[3], Zoraide Motta[4], Fabrizio Ascone[3], Lorenza Putignani[5], Alessandro Usiello[1,2,@], Loredano Pollegioni[4], Viviana Trezza[3,6], Francesco Errico[1,7,@]

[1]CEINGE Biotecnologie Avanzate "Franco Salvatore", Naples, Italy; ADM: dimaio@ceinge.unina.it; yahyavi@ceinge.unina.it; usiello@ceinge.unina.it;

[2]Department of Environmental, Biological and Pharmaceutical Sciences and Technologies, Università degli Studi della Campania "Luigi Vanvitelli", Caserta, Italy;

[3]Department of Science, Roma Tre University, Rome, Italy; valeria.buzzelli@uniroma3.it; fabrizio.ascone@uniroma3.it; viviana.trezza@uniroma3.it;

[4]"The Protein Factory 2.0", Dipartimento di Biotecnologie e Scienze della Vita, Università degli Studi dell'Insubria, Varese, Italy; zoraide.motta@uninsubria.it; Loredano.Pollegioni@uninsubria.it;

[5]Unit of Microbiomics and Unit of Research of Microbiome, Bambino Gesù Children's Hospital, IRCCS, Rome, Italy; lorenza.putignani@opbg.net;

[6]Neuroendocrinology, Metabolism and Neuropharmacology Unit, IRCCS Fondazione Santa Lucia, Rome, Italy;

[7]Dipartimento di Agraria, Università degli Studi di Napoli "Federico II", Portici, Italy; francesco.errico@unina.it.

[†]These authors contributed equally to this work

[@] **Correspondence:**

Francesco Errico: Department of Agricultural Sciences, University of Naples "Federico II", 80055, Portici, Italy; E-mail: francesco.errico@unina.it.

Alessandro Usiello: Department of Environmental, Biological and Pharmaceutical Sciences and Technologies, University of Campania "Luigi Vanvitelli", 81100, Caserta, Italy. E-mail: alessandro.usiello@unicampania.it.

Supplementary Table 1. Statistical analysis of amino acid levels in the plasma of LPS-treated rats.

Amino acid	Age	Saline (μM)			LPS (μM)			Two-way ANOVA		
		Median	IQR		Median	IQR		Factor	F (DFn, DFd)	*p* value
D-Aspartate	P35	0.1718	0.1139	0.2007	0.1139	0.06661	0.1971	Treatment<br>Age<br>Treatment x Age	-<br>-<br>-	-<br>-<br>-
	P75	-	-	-	-	-	-			
L-Aspartate	P35	2.497	1.884	4.006	2.160	1.464	4.741	Treatment<br>Age<br>Treatment x Age	0.199 (1, 18)<br>0.0006 (1, 18)<br>0.128 (1, 18)	0.6605<br>0.9793<br>0.7239
	P75	2.604	2.084	3.051	3.219	2.175	3.664			
D-Asp/total Asp (%)	P35	5.702	5.095	6.516	4.922	2.834	5.216	Treatment<br>Age<br>Treatment x Age	-<br>-<br>-	-<br>-<br>-
	P75	-	-	-	-	-	-			
L-Asparagine	P35	15.64	10.66	27.93	13.98	9.916	27.33	Treatment<br>Age<br>Treatment x Age	0.061 (1, 18)<br>0.371 (1, 18)<br>0.014 (1, 18)	0.8073<br>0.5500<br>0.9048
	P75	20.05	15.14	23.98	21.44	17.19	24.52			
D-Serine	P35	1.382	1.311	1.466	1.484	1.270	1.729	Treatment<br>Age<br>Treatment x Age	0.966 (1, 18)<br>8.412 (1, 18)<br>0.048 (1, 18)	0.3385<br>0.0095<br>0.8277
	P75	1.148	0.9573	1.172	1.275	0.9978	1.371			
L-Serine	P35	100.4	62.53	141.1	92.54	61.81	173.7	Treatment<br>Age<br>Treatment x Age	0.584 (1, 18)<br>0.002 (1, 18)<br>0.015 (1, 18)	0.4544<br>0.9629<br>0.9026
	P75	106.7	81.11	120.3	124.6	94.84	141.4			
D-Ser/total Ser (%)	P35	1.353	0.9548	2.179	1.392	1.011	2.017	Treatment<br>Age<br>Treatment x Age	0.076 (1, 18)<br>6.194 (1, 18)<br>0.003 (1, 18)	0.7849<br>0.0228<br>0.9553
	P75	1.097	0.9522	1.179	1.005	0.9238	1.104			
Glycine	P35	245.8	166.2	349.6	204.6	154.8	270.5	Treatment<br>Age<br>Treatment x Age	0.155 (1, 18)<br>3.133 (1, 18)<br>2.052 (1, 18)	0.6980<br>0.0937<br>0.1692
	P75	161.5	150.4	175.7	192.5	167.8	227.5			
L-Glutamate	P35	26.94	19.83	33.56	21.04	15.54	34.00	Treatment<br>Age<br>Treatment x Age	0.003 (1, 18)<br>3.782 (1, 18)<br>0.262 (1, 18)	0.9512<br>0.0676<br>0.6145
	P75	34.91	26.22	38.29	33.54	26.17	43.63			
L-Glutamine	P35	200.5	124.0	290.4	158.0	113.6	297.0	Treatment<br>Age<br>Treatment x Age	0.132 (1, 18)<br>2.498 (1, 18)<br>0.255 (1, 18)	0.7200<br>0.1314<br>0.6195
	P75	238.7	193.9	293.5	275.0	230.3	315.5			
L-Glutamine/ L-Glutamate	P35	7.618	6.982	8.231	7.576	7.132	8.707	Treatment<br>Age<br>Treatment x Age	1.346 (1, 18)<br>0.068 (1, 18)<br>0.286 (1, 18)	0.2612<br>0.7961<br>0.5990
	P75	7.401	6.893	7.888	7.926	6.746	9.832			

Supplementary Table 2. Statistical analysis of amino acid levels in the plasma of VPA-treated rats.

**Amino acid**	**Age**	**Saline (µM)**			**VPA (µM)**			**Two-way ANOVA**		
		**Median**	**IQR**		**Median**	**IQR**		**Factor**	**F (DFn, DFd)**	***p* value**
D-Aspartate	P40	-	-	-	-	-	-	Treatment<br>Age<br>Treatment x Age	-<br>-<br>-	-<br>-<br>-
	P90	-	-	-	-	-	-			
L-Aspartate	P40	6.652	5.463	7.743	5.726	5.315	9.035	Treatment<br>Age<br>Treatment x Age	0.756 (1, 16)<br>0.648 (1, 16)<br>1.618 (1, 16)	0.3972<br>0.4324<br>0.2215
	P90	7.275	5.709	7.976	5.173	4.665	6.444			
D-Asp/total Asp (%)	P40	-	-	-	-	-	-	Treatment<br>Age<br>Treatment x Age	-<br>-<br>-	-<br>-<br>-
	P90	-	-	-	-	-	-			
L-Asparagine	P40	13.30	10.53	21.84	19.07	13.15	29.61	Treatment<br>Age<br>Treatment x Age	0.099 (1, 16)<br>0.794 (1, 16)<br>2.129 (1, 16)	0.7569<br>0.3861<br>0.1639
	P90	16.79	11.99	22.88	11.40	8.674	20.33			
D-Serine	P40	2.585	2.278	3.167	2.892	2.299	3.296	Treatment<br>Age<br>Treatment x Age	1.595 (1, 16)<br>9.928 (1, 16)<br>0.414 (1, 16)	0.2248<br>0.0062<br>0.5287
	P90	1.821	1.702	2.265	2.506	1.956	2.603			
L-Serine	P40	103.2	77.31	151.8	106.1	78.77	161.6	Treatment<br>Age<br>Treatment x Age	0.812 (1, 16)<br>0.820 (1, 16)<br>1.457 (1, 16)	0.3808<br>0.3786<br>0.2450
	P90	128.1	84.79	144.4	72.86	56.62	112.3			
D-Ser/total Ser (%)	P40	2.904	1.503	3.641	2.735	1.433	3.963	Treatment<br>Age<br>Treatment x Age	2.282 (1, 16)<br>0.491 (1, 16)<br>1.860 (1, 16)	0.1504<br>0.4933<br>0.1915
	P90	1.718	1.399	1.968	2.748	2.107	4.044			
Glycine	P40	227.6	186.1	260.5	225.7	180.6	261.2	Treatment<br>Age<br>Treatment x Age	1.504 (1, 16)<br>10.09 (1, 16)<br>1.223 (1, 16)	0.2378<br>0.0059<br>0.2851
	P90	197.8	140.8	222.0	140.1	102.6	177.9			
L-Glutamate	P40	3.873	3.336	3.959	3.545	3.385	4.159	Treatment<br>Age<br>Treatment x Age	0.645 (1, 16)<br>0.028 (1, 16)<br>0.988 (1, 16)	0.4334<br>0.8673<br>0.3349
	P90	4.083	3.490	4.216	3.629	3.162	3.974			
L-Glutamine	P40	186.0	125.7	216.8	160.0	121.8	238.2	Treatment<br>Age<br>Treatment x Age	0.871 (1, 16)<br>0.331 (1, 16)<br>1.013 (1, 16)	0.3645<br>0.5728<br>0.3291
	P90	210.6	164.1	262.0	149.7	117.1	221.9			
L-Glutamine/ L-Glutamate	P40	48.03	37.54	54.73	46.19	35.55	57.17	Treatment<br>Age<br>Treatment x Age	0.954 (1, 16)<br>0.552 (1, 16)<br>0.873 (1, 16)	0.3432<br>0.4681<br>0.3638
	P90	51.44	47.07	62.48	42.08	36.74	55.87			

Supplementary Table 3. Statistical analysis of amino acid levels in the feces of LPS-treated rats.

Amino acid	Age	Saline (nmol/g feces)			LPS (nmol/g feces)			Two-way ANOVA		
		Median	IQR		Median	IQR		Factor	F (DFn, DFd)	*p* value
D-Aspartate	P40	25.99	17.73	31.87	22.56	14.67	37.63	Treatment<br>Age<br>Treatment x Age	0.278 (1, 36)<br>7.516 (1, 36)<br>0.073 (1, 36)	0.6007<br>0.0095<br>0.7877
	P90	11.86	8.769	18.71	12.46	7.013	19.07			
L-Aspartate	P40	59.13	46.90	95.57	72.03	58.34	97.78	Treatment<br>Age<br>Treatment x Age	0.182 (1, 36)<br>5.561 (1, 36)<br>0.912 (1, 36)	0.6715<br>0.0239<br>0.3459
	P90	43.29	32.41	84.41	47.71	34.64	64.32			
D-Asp/total Asp (%)	P40	25.47	22.07	31.56	27.25	14.72	28.59	Treatment<br>Age<br>Treatment x Age	1.685 (1, 36)<br>3.413 (1, 36)<br>0.017 (1, 36)	0.2025<br>0.0729<br>0.8962
	P90	19.74	17.40	27.80	21.19	15.32	23.60			
L-Asparagine	P40	2.477	1.876	5.301	3.240	1.444	5.997	Treatment<br>Age<br>Treatment x Age	0.116 (1, 35)<br>3.837 (1, 35)<br>0.016 (1, 35)	0.7348<br>0.0581<br>0.8995
	P90	1.855	1.233	3.149	1.611	0.7036	3.375			
D-Serine	P40	2.252	1.550	3.535	1.720	0.9791	3.194	Treatment<br>Age<br>Treatment x Age	0.008 (1, 35)<br>0.077 (1, 35)<br>0.988 (1, 35)	0.9281<br>0.7818<br>0.3270
	P90	2.028	0.9140	2.667	2.178	1.315	3.644			
L-Serine	P40	30.88	22.94	80.04	26.33	25.58	44.25	Treatment<br>Age<br>Treatment x Age	2.120 (1, 35)<br>2.001 (1, 35)<br>0.436 (1, 35)	0.1543<br>0.1660<br>0.5130
	P90	21.51	19.50	55.01	24.78	15.37	37.15			
D-Ser/total Ser (%)	P40	6.250	2.679	8.559	4.418	2.938	8.768	Treatment<br>Age<br>Treatment x Age	0.359 (1, 34)<br>1.805 (1, 34)<br>0.949 (1, 34)	0.5528<br>0.1880<br>0.3368
	P90	5.094	3.765	8.873	8.306	5.331	9.344			
Glycine	P40	41.96	13.92	148.2	73.01	30.73	121.7	Treatment<br>Age<br>Treatment x Age	1.804 (1, 34)<br>0.085 (1, 34)<br>0.110 (1, 34)	0.1881<br>0.7718<br>0.7416
	P90	69.13	27.17	133.5	48.81	5.872	84.69			
L-Glutamate	P40	308.7	194.5	421.6	244.0	211.4	387.0	Treatment<br>Age<br>Treatment x Age	0.006 (1, 36)<br>0.554 (1, 36)<br>3.1e-005 (1, 36)	0.9385<br>0.4614<br>0.9956
	P90	264.5	141.6	420.0	225.1	177.1	445.0			
L-Glutamine	P40	29.01	12.10	38.67	23.35	18.98	43.02	Treatment<br>Age<br>Treatment x Age	0.020 (1, 36)<br>3.017 (1, 36)<br>0.029 (1, 36)	0.8875<br>0.0910<br>0.8645
	P90	18.84	10.52	31.86	19.23	16.69	31.94			
L-Glutamine/ L-Glutamate	P40	0.08979	0.05702	0.1229	0.08913	0.07298	0.1142	Treatment<br>Age<br>Treatment x Age	0.107 (1, 36)<br>2.181 (1, 36)<br>0.185 (1, 36)	0.7449<br>0.1484<br>0.6695
	P90	0.06673	0.05405	0.08768	0.08946	0.05688	0.1125			

Supplementary Table 4. Statistical analysis of amino acid levels in the feces of VPA-treated rats.

Amino acid	Age	Saline (nmol/g feces)			VPA (nmol/g feces)			Two-way ANOVA		
		Median	IQR		Median	IQR		Factor	F (DFn, DFd)	*p* value
D-Aspartate	P30	13.73	8.786	17.19	11.49	8.504	28.53	Treatment<br>Age<br>Treatment x Age	3.235 (1, 36)<br>0.022 (1, 36)<br>0.0001 (1, 36)	0.0805<br>0.8827<br>0.9906
	P80	12.03	4.779	16.88	14.85	8.253	46.86			
L-Aspartate	P30	39.89	29.47	78.19	48.51	25.78	76.10	Treatment<br>Age<br>Treatment x Age	0.928 (1, 36)<br>0.329 (1, 36)<br>0.004 (1, 36)	0.3417<br>0.5694<br>0.9448
	P80	39.32	21.46	58.33	44.32	26.03	98.28			
D-Asp/total Asp (%)	P30	21.77	17.69	27.44	27.49	16.14	31.25	Treatment<br>Age<br>Treatment x Age	2.629 (1, 36)<br>0.638 (1, 36)<br>0.619 (1, 36)	0.1137<br>0.4296<br>0.4363
	P80	21.93	16.19	29.05	30.98	20.77	34.92			
L-Asparagine	P30	4.655	3.166	7.786	4.787	2.611	9.676	Treatment<br>Age<br>Treatment x Age	0.893 (1, 36)<br>1.030 (1, 36)<br>0.052 (1, 36)	0.3508<br>0.3170<br>0.8197
	P80	2.979	2.212	5.578	4.494	2.313	7.610			
D-Serine	P30	1.229	0.6118	2.200	1.269	0.7242	3.061	Treatment<br>Age<br>Treatment x Age	0.807 (1, 36)<br>0.344 (1, 36)<br>0.106 (1, 36)	0.3748<br>0.5607<br>0.7460
	P80	1.520	0.8087	2.786	2.155	1.115	4.149			
L-Serine	P30	22.86	16.62	61.44	26.84	15.46	46.14	Treatment<br>Age<br>Treatment x Age	0.001 (1, 36)<br>2.039 (1, 36)<br>1.292 (1, 36)	0.9719<br>0.1619<br>0.2632
	P80	21.01	9.348	27.91	27.21	11.87	45.15			
D-Ser/total Ser (%)	P30	7.127	1.011	10.92	5.148	3.183	8.061	Treatment<br>Age<br>Treatment x Age	0.345 (1, 36)<br>3.086 (1, 36)<br>0.060 (1, 36)	0.5602<br>0.0875<br>0.8070
	P80	7.456	5.836	11.67	8.751	7.149	10.53			
Glycine	P30	129.0	11.16	412.6	161.9	21.77	358.6	Treatment<br>Age<br>Treatment x Age	0.016 (1, 36)<br>4.250 (1, 36)<br>0.134 (1, 36)	0.8999<br>0.0465<br>0.7161
	P80	54.86	26.70	127.4	76.82	29.30	193.2			
L-Glutamate	P30	291.7	160.4	420.1	359.9	105.2	710.0	Treatment<br>Age<br>Treatment x Age	0.327 (1, 36)<br>0.469 (1, 36)<br>0.426 (1, 36)	0.5708<br>0.4976<br>0.5178
	P80	256.6	199.4	460.0	280.3	200.3	651.7			
L-Glutamine	P30	17.41	13.25	36.58	20.93	12.97	41.12	Treatment<br>Age<br>Treatment x Age	1.124 (1, 36)<br>6e-005 (1, 36)<br>0.953 (1, 36)	0.2961<br>0.9938<br>0.3354
	P80	14.90	12.84	29.17	25.28	12.94	53.27			
L-Glutamine/ L-Glutamate	P30	0.08487	0.06994	0.1026	0.07781	0.05587	0.1271	Treatment<br>Age<br>Treatment x Age	0.719 (1, 36)<br>2.010 (1, 36)<br>0.773 (1, 36)	0.4019<br>0.1649<br>0.3850
	P80	0.05776	0.03090	0.08157	0.07228	0.05163	0.09342			

Supplementary Table 5. Statistical analysis of DASPO, DAAO and SR activity and predicted enzyme concentrations in the prefrontal cortex and dorsal striatum of LPS-treated rats.

Enzyme	Tissue	Age	Saline (µU/µg protein) [ng enzyme/µg protein]		LPS (µU/µg protein) [ng enzyme/µg protein]		Two-way ANOVA		
			Mean	SEM	Mean	SEM	Factor	F (DFn, DFd)	*P* value
DASPO	PFC	P35	0.0984<br>[0.00190]	0.0078<br>[0.00015]	0.1089<br>[0.00211]	0.0067<br>[0.00013]	Treatment<br>Age<br>Treatment x Age	2.19 (1, 20)<br>5.65 (1, 20)<br>7.16 (1, 20)	0.1540<br>**0.0276**<br>**0.0144**
		P75	0.1011<br>[0.00196]	0.0125<br>[0.00024]	0.0649<br>[0.00125]	0.0063<br>[0.00012]			
	STR	P35	0.0510<br>[0.000986]	0.0053<br>[0.00010]	0.0468<br>[0.000906]	0.0045<br>[0.00009]	Treatment<br>Age<br>Treatment x Age	2.699 (1, 19)<br>7.130 (1, 19)<br>0.713 (1, 19)	0.1169<br>**0.0149**<br>0.4089
		P75	0.0692<br>[0.00134]	0.0064<br>[0.00012]	0.0563<br>[0.00109]	0.0047<br>[0.00009]			
DAAO	PFC	P35	0.0076<br>[0.00019]	0.0015<br>[0.00004]	0.0091<br>[0.00023]	0.0008<br>[0.00002]	Treatment<br>Age<br>Treatment x Age	0.14 (1, 20)<br>15.9 (1, 20)<br>1.77 (1, 20)	0.7079<br>**0.0007**<br>0.1981
		P75	0.0162<br>[0.00040]	0.0025<br>[0.00006]	0.0134<br>[0.00033]	0.0013<br>[0.00003]			
	STR	P35	0.0027<br>[0.000067]	0.0015<br>[0.000038]	0.00275<br>[0.000068]	0.0016<br>[0.000039]	Treatment<br>Age<br>Treatment x Age	0.166 (1, 19)<br>22.44 (1, 19)<br>0.171 (1, 19)	0.6886<br>**0.0001**<br>0.6835
		P75	0.0154<br>[0.000380]	0.0026<br>[0.000064]	0.0134<br>[0.00033]	0.0035<br>[0.000087]			
SR	PFC	P35	1.26<br>[0.574]	0.09<br>[0.043]	1.20<br>[0.543]	0.08<br>[0.039]	Treatment<br>Age<br>Treatment x Age	5.259 (1, 20)<br>0.636 (1, 20)<br>2.309 (1, 20)	**0.0328**<br>0.4345<br>0.1443
		P75	1.46<br>[0.672]	0.05<br>[0.026]	1.13<br>[0.515]	0.10<br>[0.045]			
	STR	P35	1.11<br>[0.502]	0.23<br>[0.107]	1.44<br>[0.652]	0.32<br>[0.145]	Treatment<br>Age<br>Treatment x Age	3.083 (1, 19)<br>1.526 (1, 19)<br>0.511 (1, 19)	0.0952<br>0.2318<br>0.4833
		P75	1.27<br>[0.578]	0.16<br>[0.071]	2.05<br>[0.934]	0.44<br>[0.198]			

Supplementary Table 6. Statistical analysis of DASPO, DAAO and SR activity and predicted enzyme concentrations in the prefrontal cortex and dorsal striatum of VPA-treated rats.

**Enzyme**	**Tissue**	**Age**	**Saline (µU/µg protein) [ng enzyme/µg protein]**		**VPA (µU/µg protein)[ng enzyme/µg protein]**		**Two-way ANOVA**		
			**Mean**	**SEM**	**Mean**	**SEM**	**Factor**	**F (DFn, DFd)**	***P* value**
DASPO	PFC	P40	0.0998 [0.00193]	0.0090 [0.00017]	0.0903 [0.00174]	0.0090 [0.00018]	Treatment<br>Age<br>Treatment x Age	0.057 (1, 20)<br>0.599 (1, 20)<br>0.878 (1, 20)	0.8132<br>0.4480<br>0.3600
		P90	0.0860 [0.00166]	0.0072 [0.00014]	0.0917 [0.00177]	0.0067 [0.00013]			
	STR	P40	0.0693 [0.00134]	0.0056 [0.00011]	0.0782 [0.00151]	0.0047 [0.00009]	Treatment<br>Age<br>Treatment x Age	2.087 (1, 19)<br>0.0001 (1, 19)<br>0.164 (1, 19)	0.1649<br>0.9907<br>0.6905
		P90	0.0658 [0.00127]	0.0091 [0.00018]	0.0815 [0.00158]	0.0124 [0.00024]			
DAAO	PFC	P40	0.0145 [0.00036]	0.0002 [0.00001]	0.0131 [0.00032]	0.0007 [0.00002]	Treatment<br>Age<br>Treatment x Age	3.928 (1, 20)<br>32.93 (1, 20)<br>0.128 (1, 20)	0.0614<br>**<0.0001**<br>0.7240
		P90	0.0100 [0.00025]	0.0011 [0.00003]	0.0081 [0.00020]	0.0010 [0.00002]			
	STR	P40	0.0142 [0.00036]	0.0008 [0.00002]	0.0158 [0.00039]	0.0030 [0.00007]	Treatment<br>Age<br>Treatment x Age	0.1405 (1, 19)<br>7.866 (1, 19)<br>0.1020 (1, 19)	0.7119<br>**0.0113**<br>0.7530
		P90	0.0082 [0.00020]	0.0027 [0.00007]	0.0083 [0.00021]	0.0025 [0.00006]			
SR	PFC	P40	1.58 [0.718]	0.12 [0.056]	1.07 [0.487]	0.12 [0.056]	Treatment<br>Age<br>Treatment x Age	7.22 (1, 20)<br>13.1 (1, 20)<br>4.55 (1, 20)	**0.0142**<br>**0.0017**<br>0.0454
		P90	0.97 [0.442]	0.08 [0.035]	0.92 [0.415]	0.09 [0.041]			
	STR	P40	1.66 [0.755]	0.14 [0066]	2.11 [0.956]	0.26 [0.118]	Treatment<br>Age<br>Treatment x Age	2.67 (1, 19)<br>22.1 (1, 19)<br>1.00 (1, 19)	0.1185<br>**0.0002**<br>0.3295
		P90	1.04 [0.473]	0.11 [0.049]	1.15 [0.5212]	0.08 [0.037]			

Supplementary Table 7. Statistical analysis of DASPO, DAAO and SR activity and predicted enzyme concentrations in the prefrontal cortex and dorsal striatum of *Fmr1-Δexon 8* rats.

Enzyme	Tissue	Wild-type (μU/μg protein) [ng enzyme/μg protein]		*Fmr1-Δexon 8* (μU/μg protein) [ng enzyme/μg protein]		Unpaired t-test	
		**Mean**	**SEM**	**Mean**	**SEM**	**t, df**	***P* value**
DASPO	PFC	0.0380 [0.000736]	0.0040 [0.000077]	0.0280 [0.000541]	0.0072 [0.000139]	1.224, 10	0.1246
	HIPP	0.0401 [0.000776]	0.0082 [0.000160]	0.0383 [0.000741]	0.0032 [0.000063]	0.209, 10	0.4194
	NAc	0.0146 [0.000283]	0.0031 [0.000060]	0.0141 [0.000273]	0.0033 [0.000063]	0.117, 10	0.4547
	STR	0.0252 [0.000488]	0.0104 [0.000200]	0.0173 [0.000335]	0.0027 [0.000053]	0.736, 10	0.2392
DAAO	PFC	0.0025 [0.000061]	0.0005 [0.000011]	0.0027 [0.000066]	0.0008 [0.000019]	0.238, 10	0.4084
	HIPP	0.0017 [0.000043]	0.0005 [0.000011]	0.0037 [0.000091]	0.0004 [0.000010]	3.151, 10	**0.0052**
	NAc	0.0012 [0.000029]	0.0007 [0.000017]	0.0023 [0.000056]	0.0006 [0.000015]	1.196, 10	0.1296
	STR	0.0031 [0.000077]	0.0013 [0.000032]	0.0025 [0.000060]	0.0008 [0.000019]	0.438, 10	0.3355
SR	PFC	0.464 [0.211]	0.098 [0.044]	0.518 [0.235]	0.115 [0.052]	0.358, 10	0.3638
	HIPP	0.619 [0.282]	0.109 [0.050]	0.479 [0.218]	0.086 [0.039]	1.011, 10	0.1680
	NAc	0.754 [0.343]	0.140 [0.063]	0.737 [0.335]	0.169 [0.077]	0.076, 10	0.4703
	STR	0.646 [0.294]	0.114 [0.052]	0.486 [0.221]	0.117 [0.053]	0.981, 10	0.1749
